# Mass spectrometry of dendrimers

**DOI:** 10.1002/mas.21876

**Published:** 2024-03-19

**Authors:** McKenna J. Redding, Scott M. Grayson, Laurence Charles

**Affiliations:** ^1^ Department of Chemistry Tulane University New Orleans Louisiana USA; ^2^ Aix Marseille Université, CNRS Institut de Chimie Radicalaire Marseille France

**Keywords:** dendrimers, ion mobility spectrometry, ionization, mass analysis, tandem mass spectrometry

## Abstract

Mass spectrometry (MS) has become an essential technique to characterize dendrimers as it proved efficient at tackling analytical challenges raised by their peculiar onion‐like structure. Owing to their chemical diversity, this review covers benefits of MS methods as a function of dendrimer classes, discussing advantages and limitations of ionization techniques, tandem mass spectrometry (MS/MS) strategies to determine the structure of defective species, as well as most recently demonstrated capabilities of ion mobility spectrometry (IMS) in the field. Complementarily, the well‐defined structure of these macromolecules offers major advantages in the development of MS‐based method, as reported in a second section reviewing uses of dendrimers as MS and IMS calibration standards and as multifunctional charge inversion reagents in gas phase ion/ion reactions.

## 1 INTRODUCTION

**Figure 1:**
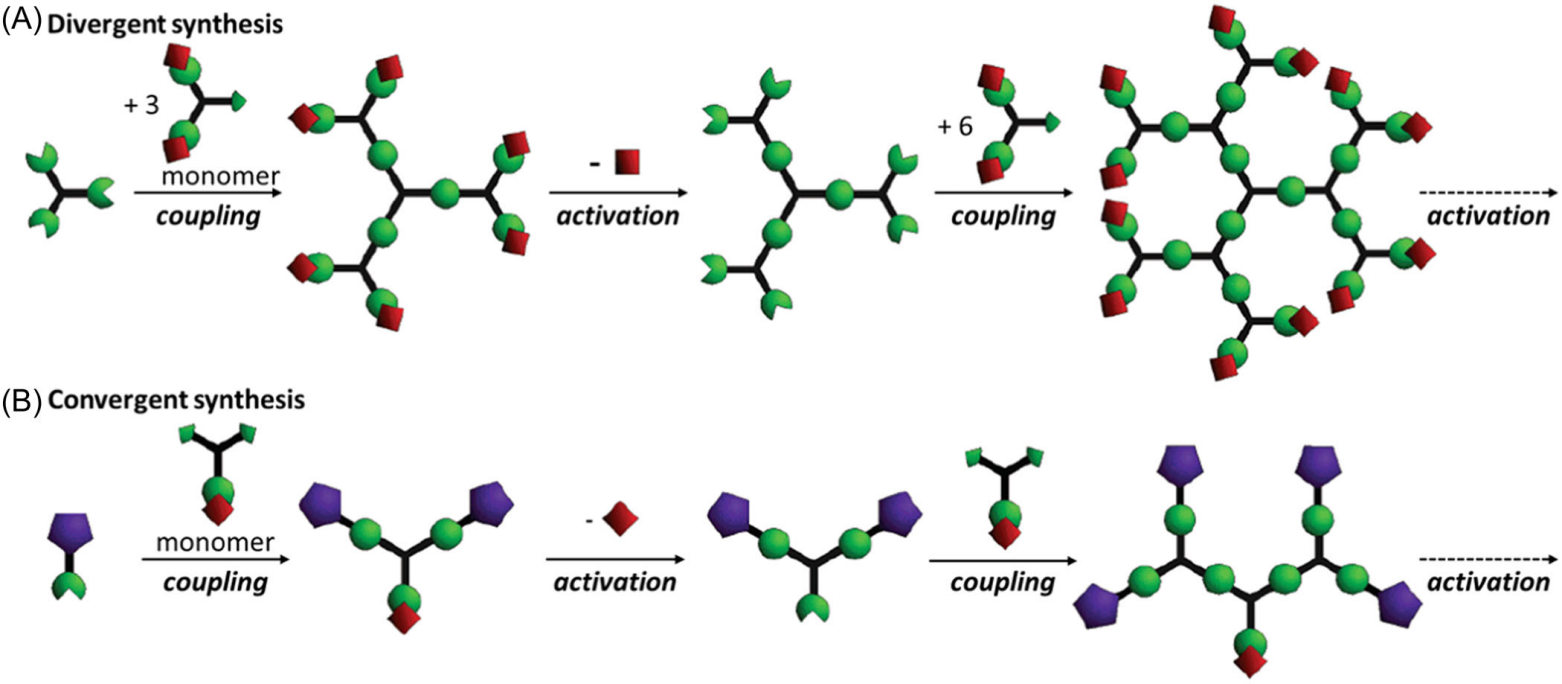
Schematic representation of dendrimer synthesis according to (A) divergent and (B) convergent processes. Adapted from Grayson and Frechet (2001), with permission from the American Chemical Society. [Color figure can be viewed at wileyonlinelibrary.com]

Dendrimers are globular synthetic macromolecules with very narrow molecular distribution (Tomalia & Frechet, 2001), characterized by a particular architecture composed of three distinct domains: a central core (or a focal point), arms emanating from the core and terminal functional groups (Figure 1). The central core can be a single atom or a small chemical moiety having at least two identical functions. Arms are made of branches comprised of repeating units with at least one junction, and their repetition results in a series of radially concentric layers named generations (Gi). External terminations can be further functionalized to give specific properties to the dendrimer. These macromolecules are typically prepared via two main synthetic pathways (Grayson & Frechet, 2001). Divergent processes (Figure 1A) involve initial reaction of the monomer unit with a polyfunctional core and continues outward by the repetition of coupling and activation steps, the number of which defines the dendrimer generation (Tomalia et al., 1985). The divergent approach enables preparation of dendrimers of very high generation but its main drawback remains the production of defective molecules due to incomplete reactions of terminal groups, the number of which increases rapidly with the generation. Use of excess reagents to prevent formation of these by‐products further complicates dendrimer purification, therefore very few of the divergent syntheses are pure. In contrast, convergent pathways (Figure 1B) involve the synthesis of small structures named dendrons, that will ultimately constitute the outside of the dendrimer, followed by their coupling to a central (focal) core (Hawker & Frechet, 1990). The inward progress of such pathways limits the number of reactions and hence occurrence of side‐products. Moreover, even though by‐products missing one or more dendritic wedges are generated, they can be readily removed from the dendrimer sample by purification thanks to their large structural differences compared to the perfect molecule. However, the convergent strategy requires column chromatography for each generation and the reactions of very large dendrimers reduce the overall final yield.

Since their topology and functionality can be controlled by the synthetic scheme, dendrimers with predefined properties can be prepared for a variety of applications in different fields where they can act as nano‐scale objects (Balzani et al., 2008), catalysts (Zhao & Crooks, 1999) or cargos in drug delivery (Caminade & Turrin, 2014). As a result, detailed structural characterization of these macromolecules is mandatory but there is a critical need for techniques offering capabilities to address analytical challenges raised by their peculiar architecture. Nuclear magnetic resonance (NMR) is very popular for dendrimer analysis, particularly for species containing NMR‐active nuclei such as phosphorus, silicon, or fluorine. NMR is often used to check the conversion of a particular reaction during dendrimer synthesis but signals corresponding to impurities can easily go unnoticed, either due to their low intensity compared to a large generation dendrimer or coincidence with other signals. Moreover, because the building blocks are the same in each dendrimer shell but are located in different microenvironments, NMR spectra of high generations usually exhibit broad signals which cannot be precisely assigned. Similarly, detection of defects by infrared spectroscopy is increasingly difficult in higher generations because signals corresponding to single defect are very weak. Separation techniques like chromatography and electrophoresis are useful to separate perfect dendrimers from their defective congeners but cannot provide information about the nature of defects. The unusual viscosity of dendrimers, their interaction with the stationary phase as well as the lack of appropriate calibrants makes gel permeation chromatography (GPC) ineffective for accurate determination of their molecular weight. As compared to the previously described techniques, mass spectrometry (MS) has the ability to separate individual components of a mixture, based on their mass‐to‐charge ratio (*m/z*) as ions in the gas phase. Using soft ionization techniques to produce intact ions from nonvolatile macromolecules, MS allows precise mass measurement of dendritic species and distinction of minute amounts of structurally related impurities from perfect structures. Tandem mass spectrometry (MS/MS) experiments can further be implemented for detailed structural characterization of impurities while the most recent instrumentations in ion mobility spectrometry (IMS) offer unique insights on the shape and conformational changes of dendrimers.

The sole article reviewing main analytical techniques used for characterization of dendrimers was published in 2005 (Caminade et al., 2005) and included a small section on MS with a total of 15 references. Since then, MS has become a routine tool in dendrimer studies and some review articles dedicated to MS of synthetic polymers include a section with main contributions in the field (Weidner & Trimpin, 2010). The present article is mainly focused on MS of dendrimers and specific challenges faced during their analysis. The first section describes how MS has been usefully employed for dendrimer characterization. It first includes the ionization step, discussing experimental conditions as well as advantages and limitations of ionization techniques as a function of dendrimer classes. Then, investigations on dendrimer fragmentation and structural characterization of defects by MS/MS are reported, before introducing most recently demonstrated capabilities of IMS in the field of dendrimers. The second section reviews benefits of using dendrimers to develop MS methods. Indeed, the peculiar structure of these well‐defined macromolecules have found relevant applications in analytical chemistry, as reported in two main axes: their use as standards for mass calibration (in MS) or size calibration (in IMS) on the one hand, and as reagents for gas‐phase ion–ion reactions on the other hand. For readers unfamiliar with MS, all acronyms related to instrumentation and experiments are defined throughout the text but also listed in Table 1.

**Table 1:** Acronyms related to mass spectrometry instrumentation and experiments.

CID	collision‐induced dissociation
DMA	differential mobility analysis
ECD	electron capture dissociation
EDD	electron detachment dissociation
ESI	electrospray ionization
FAB	fast atom bombardment
FT‐ICR	Fourier transform ion cyclotron resonance
IMS	ion mobility spectrometry
IRMPD	infrared multiphoton dissociation
L‐SIMS	liquid secondary ion mass spectrometry
LDI	laser desorption/ionization
MALDI	matrix‐assisted laser desorption/ionization
MS	mass spectrometry
MS/MS	tandem mass spectrometry
oaTOF	orthogonal acceleration time of flight
PSD	postsource decay
SALDI	surface‐assisted laser desorption/ionization
SID	surface‐induced dissociation
SORI‐CAD	sustained off‐resonance irradiation collisional activation dissociation
TOF	time of flight
TWIMS	traveling wave ion mobility spectrometry

## 2 MS FOR DENDRIMERS

### 2.1 Ionization of dendrimers for MS

Ionization of dendrimers as intact gas phase ions requires the use of techniques developed for nonvolatile (macro)molecules. Although fast atom bombardment (FAB) (Gonzalez et al., 2000; Hawker & Frechet, 1990; Twyman et al., 1994) or liquid secondary ion mass spectrometry (L‐SIMS) (Lau et al., 1995) have been employed in a few early studies to produce molecular ions or protonated molecules of very small dendrimers (MW < 2 kDa), the most common techniques used nowadays for dendritic species are matrix‐assisted laser desorption/ionization (MALDI) (Karas & Hillenkamp, 1988; Tanaka et al., 1988) and electrospray ionization (ESI) (Fenn et al., 1990). A vast majority of studies employs MALDI as this technique minimizes the formation of multiply charged species and hence produces less complex mass spectra compared to ESI. MALDI mass data indeed permit to distinguish, at a glance, monodisperse dendrimer samples from those containing defective molecules. Yet, MALDI is not reputed as soft as ESI and may induce, either upon photo‐ or collision‐activation, formation of dendrimer fragments that need to be distinguished from synthesis by‐products actually present in dendrimer samples. The use of ESI in dendrimer studies is also dictated by instrumental configuration. Determination of ion elemental composition from their accurate mass measurements requires high‐resolution MS, as enabled by orthogonal acceleration time‐of‐flight (oa‐TOF) or Fourier‐transform ion cyclotron resonance (FT‐ICR) mass analyzers, most often found in instruments equipped with ESI. Similarly, commercial mass spectrometers that offer MS/MS or IMS capabilities are most often equipped with an ESI source. Spectral complexity introduced by multiple charging in ESI can be circumvented by plotting data in the mass domain upon charge deconvolution enabled by a variety of dedicated software. Yet, multiple charging of dendrimers in ESI can also be usefully employed to decipher relevant information regarding ion conformation from both the neat number of charges and charge state distribution. Last but not least, ESI is an ideal interface for coupling MS with liquid chromatography since this ionization process relies on a flowing liquid.

This first section deals with gas phase production of dendrimer ions; yet, it does not aim at producing an exhaustive inventory of all published studies mentioning MS of dendrimers but, instead, to focus on those reports containing detailed description of ionization conditions (in MALDI and/or in ESI) and of any issues related to this crucial step, as a function of dendrimer families. In particular, the most critical parameter affecting the success of MALDI is undoubtedly proper choice of the matrix. Although the MALDI process is still not fully rationalized, the matrix should be selected as a function of the analyte chemistry. Owing to the wide variety of dendrimers, no universal matrix is available and matrix selection is performed either by trial and error or based on prior reported literature. All acronyms used herein to designate matrices are listed in Table 2. Quality of MALDI mass data also relies on the matrix‐to‐analyte molar ratio which often has to be optimized as a function of dendrimer generation. Solvents used for sample preparation are another key parameter, obviously in ESI since the flowing liquid is part of the ionization process and highly influences ion yield, but also in MALDI despite the fact that final samples are dry solids. Indeed, a vast majority of studies reporting MALDI‐MS of dendrimers use the classical “dried droplet” method for sample preparation, where a binary solution is first prepared by mixing proper quantities of individual liquid solutions of the analyte and of the matrix, before an aliquot (~1 μL) is deposited onto the MALDI plate and left to evaporate. Although solvents used to prepare MALDI samples are no longer present in the laser‐irradiated solid, they can affect MALDI sample homogeneity and hence mass spectral quality. This was, for example, reported by Zhao et al. while using chloroform, a good solvent for the studied dendrimer but a poor solvent for the CHCA matrix, to prepare MALDI samples: scanning electron microscopy revealed that CHCA crystals of different size were formed upon solvent evaporation, leading to spectra with mass drift and decreased resolution (Zhao et al., 2003). Solvent selection is also particularly crucial when sample preparation includes addition of a salt (most often better solubilized in polar media) to favor cation adduction for dendrimers with low proton affinity. For example, dendrimers containing oxygenated groups exhibit high affinity towards alkali and readily ionize as [M + Na]^+^ and [M + K]^+^ adducts, taking advantage of sodium and potassium present at trace levels in matrices or in solvents stored in glass bottles. Yet, cationizing efficiency can be improved by supplementing ESI or MALDI samples with an alkali salt, which also helps to promote the formation of a single adduct and hence prevents signal dilution over multiple ionic forms.

**Table 2:** Acronym and full name of matrices mentioned in this section.

1,8,9‐ACT	1,8,9‐anthracenetriol
5‐CSA	5‐chlorosalicylic acid
2,5‐DHB	2,5‐dihydroxybenzoic acid
3,5‐DHB	3,5‐dihydroxybenzoic acid
7‐HC	7‐hydroxycoumarin
9‐NA	9‐nitroanthracene
ATT	6‐aza‐2‐thiothymine
CHCA	α‐cyano‐4‐hydroxycinnamic acid
DAN	1,5‐diaminonaphthalene
DCTB	2‐[(2*E*)‐3‐(4‐*tert*‐butylphenyl) ‐2‐methylprop‐2‐enylidene] malononitrile
DHAP	dihydroxyacetophenone
DIT	dithranol
FA	ferulic acid
GFA	galvinoxyl free radical
HABA	2‐(4‐hydroxyphenylazo) benzoic acid
IAA	*trans*‐indoleacrylic acid
MBT	mercaptobenzothiazole
nor‐HM	nor‐harmane
*t*‐RA	*trans*‐retinoic acid
SA	sinapinic acid
THAP	2,4,6‐trihydroxyacetophenone

The experimental conditions as well as advantages and limitations of MALDI and ESI are reported for the MS of dendrimers and will be inventoried hereafter for the significant main classes. We chose to limit this review to organic dendrimers as obtained at the end of the synthetic process, hence excluding metallo‐dendrimers, post‐functionalized species and supramolecular assemblies.

#### 2.1.1 Hydrocarbon dendrimers

**Figure 2:**
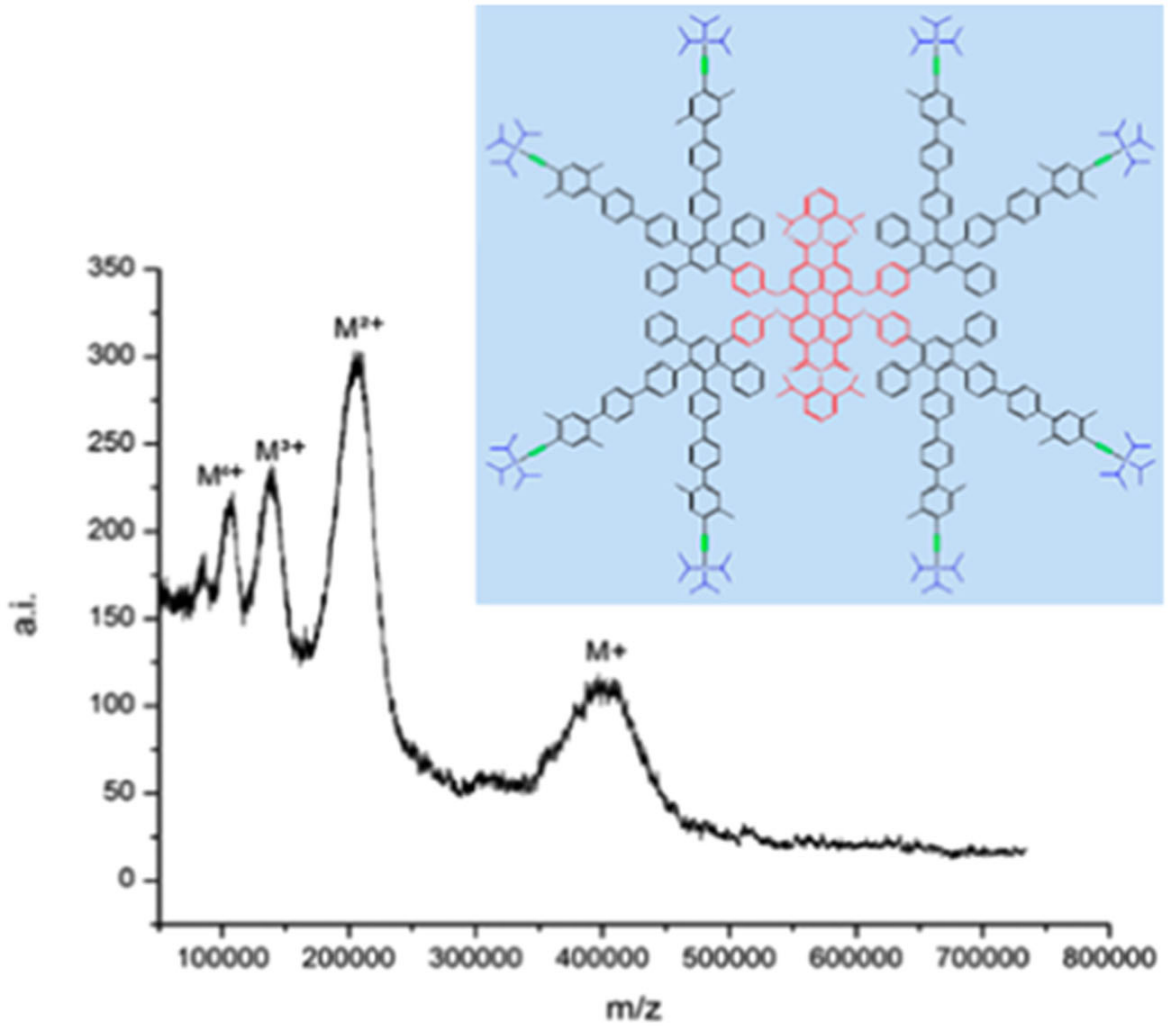
Matrix‐assisted laser desorption/ionization‐time of flight mass spectrum of giant polyphenylene dendrimer G7 (422 kDa) built in divergent synthesis from the G1 structure shown in inset, using 2‐[(2*E*)‐3‐(4‐*tert*‐butylphenyl)‐2‐methylprop‐2‐enylidene] malononitrile (DCTB) as matrix and THF as solvent. Reprinted from Rader et al. (2014), with permission from the American Chemical Society. [Color figure can be viewed at wileyonlinelibrary.com]

The earliest study employing MALDI for MS analysis of dendrimers was reported in 1994. It targeted the challenging phenylacetylene dendrimers, that are mainly soluble in apolar solvents (hence escaping ESI) and do not contain any heteroatoms to favor cation adduction (Xu et al., 1994). While direct infrared laser desorption enabled accurate mass measurements of protonated species for the smallest G0 (715.2 Da) and G1 (1652.4 Da) dendrimers by FT‐ICR‐MS, AgNO_3_ had to be added in a so‐called laser desorption‐chemical ionization approach to successfully ionize the G3 molecule (3526.7 Da) as a silver adduct. Yet, assistance of a matrix, namely *t‐*RA (with matrix‐to‐analyte molar ratio of 550:1), was required for the largest G4 (7276.3 Da) and G5 (14775.2 Da) species to be detected in linear mode TOF. In contrast to other poorly or not efficient matrices tested in UV‐MALDI, *t‐*RA is highly soluble in methylene chloride and was hence well suited to hydrocarbon dendrimer analysis. Yet, first it was photoisomerized upon exposure to fluorescent light for 72 h to best act as a MALDI matrix (Walker et al., 1994). This sample preparation was further employed for mass analysis of much larger phenylacetylene dendrimers synthesized via an accelerated convergent process using 15‐mer dendron reagents (Kawaguchi et al., 1995). Using *t‐*RA with matrix‐to‐analyte molar ratios from 5300:1 to 17,300:1 permitted to desorb macromolecules in the 35–50 kDa range: detection of the 195‐, 210‐, 225‐, and 240‐mers revealed an incomplete coupling of 15‐mer dendrons, which was hence optimized to achieve a nearly monodisperse 255‐mer sample. These pioneering works also revealed dendrimer clustering, which became more pronounced as the laser fluence was increased. This aggregation phenomenon was later studied for polyphenylene dendrimers by Müllen and coworkers, who employed a superconductive cryodetector to extend sensitivity into the MDa range and ion charge state to be determined based on their kinetic energy (Wenzel et al., 2005). The strong solvophobic interactions driving dendrimer aggregation during sample preparation could not be decreased by simply lowering the matrix polarity, for example, using 2‐[(2*E*)‐3‐(4‐*tert*‐butylphenyl)‐2‐methylprop‐2‐enylidene] malononitrile (DCTB) instead of dithranol (DIT) (Clark et al., 2007a, 2007b). Instead, extent of clustering could be minimized by enhancing sample dilution in THF (Rader et al., 2014), which enabled successful MALDI of polyphenylene dendrimers up to the MDa range (Figure 2). Using DCTB, these giant macromolecules were observed over multiple charge states (Nguyen et al., 2013; Rader et al., 2014). Charge deconvolution of these MALDI data permitted to demonstrate that dendrimers were observed as perfect structures up to G4 (57 kDa) while an increasing number of missing branches with increasing the generation number accounted for slight deviations from theoretical masses observed from G5 (116 kDa) up to G9 (1.5 MDa). Beside these major achievements, issues were also reported during MALDI of those polyphenylene dendrimers bearing tetraphenylmethane moieties, which UV absorbance causes fragmentation regardless of the matrix used (Andreitchenko et al., 2005; Urzua et al., 2016).

#### 2.1.2 Polyamidoamine (PAMAM) dendrimers

The most famous dendrimers are undoubtedly PAMAM, also known as Starburst polymers. Since they are commercially available, PAMAMs are often used as dendrimer models to establish performance of analytical methods. In MS, they have long been preferentially ionized by ESI before successful MALDI was achieved by different groups. Taking advantage of highly charged species produced in ESI, Tomalia and coworkers have reported MS of a G4 PAMAM (10,632 Da) (Kallos et al., 1991), then used a quadrupole mass analyzer with an extended *m/z* range for the detection of up to G10 (Schwartz et al., 1995) before capitalizing on the much higher resolving power of FT‐ICR to achieve detailed characterization of a G1–G5 PAMAM series (Tolic et al., 1997). These pioneering studies showed that a variety of additives such as formic acid (0.1%), acetic acid (0.1% or 5%), or ammonium acetate (10 mM) could be safely employed to favor protonation of PAMAM with no adduct formation. Alternatively, adduction of Ag^+^ cation was shown to readily occur during ESI of PAMAM dendrimer in a 3:1 methanol/water mixture supplemented with 10 μM of silver perchlorate (Fan et al., 2005). Most reported studies used water/methanol (50/50 or 90/10) to solubilize these polar analytes although acetonitrile was preferred as the organic solvent when coupling ESI‐MS with reversed‐phase liquid chromatography (Lloyd et al., 2016; Ucles et al., 2013). Multiple charging in ESI was also usefully employed to reveal an even distribution of charges at the surface of PAMAM globular structures as their average charge state was observed to vary linearly with M^2/3^ (Schwartz et al., 1995).

**Figure 3:**
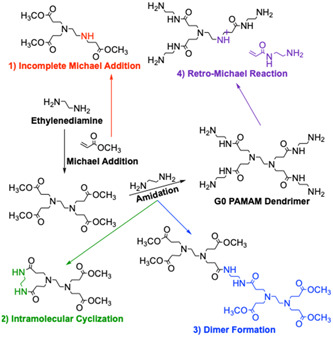
Possible structural defects generated by the different side reactions in polyamidoamine dendrimer synthesis. [Color figure can be viewed at wileyonlinelibrary.com]

Beside prominent signals of the perfect species, mass spectra of PAMAM dendrimers often exhibit additional minor signals, the number of which rapidly increases with dendrimer generation. The divergent synthetic strategy employed for the production of PAMAM may yield defective side products. Production of PAMAM typically consists of an iterative sequence of two reactions, a Michael addition of methyl acrylate onto secondary amine end‐groups followed by amidation of the resulting methyl ester derivatives with ethylene diamine to regenerate terminal amines (Tomalia et al., 1990). As depicted in Figure 3, subquantitative success of the Michael addition (pathway 1) or retro‐Michael reaction (pathway 4) leads to the “missing arm” impurity, revealed by a 114 Da mass defect. Formation of dimers (pathway 3) and intramolecular cyclization leading to “molecular loop” (pathway 2, 60 Da mass defect) during the amidation step are due to the bifunctional character of ethylene diamine. As the generation grows, reactions involving these defective forms of the dendrimer give rise to new impurities, and hence lead to increasingly heterogeneous samples. Assuming similar ionization yields of all species regardless of their size and structure, mass data can be used to qualify sample polydispersity (Kallos et al., 1991), as calculated by *M*_w_/*M*_n_, with *M*_n_ the number average molecular weight (*M*_n_ = Σ*N*_i_ *M*_i_/Σ*N*_i_) and *M*_w_ the weight average molecular weight (*M*_w_ = Σ*N*_i_M_i_^2^/Σ*N*_i_ *M*_i_), where *N*_i_ is the abundance of molecules of mass *M*_i_.

An ESI‐related issue was reported by Maire and Lange while analyzing a small ethylenediamine‐core PAMAM in the negative ion mode (Maire & Lange, 2010). Unexpected ions were observed at +12 *m/z* compared to the targeted [G1–H]^–^ whereas no corresponding signal was noticed in the positive mode. The authors found that this artefact was due to corona discharge occurring in the negative mode and producing reactive oxygen species that would promote partial decomposition of methanol into oxidized by‐products able to react with one of the numerous NH_2_ end‐group of PAMAM to form an imine. Yet, it should be acknowledged that this species was mainly observed in very unusual experimental conditions for mass analysis of PAMAM (i.e., negative ion mode and very high voltage applied to corroded capillary emitter). Interestingly, the same “+12 *m/z* species” was later reported in ESI mass spectra recorded in the positive ion mode for ethylenediamine‐core PAMAM dendrimer (Tintaru et al., 2015). In this latter case, it was demonstrated that modification of one ethylene diamine end‐group into a cyclic imidazolidine moiety was induced by traces of formaldehyde in methanol used as the synthesis medium.

**Figure 4:**
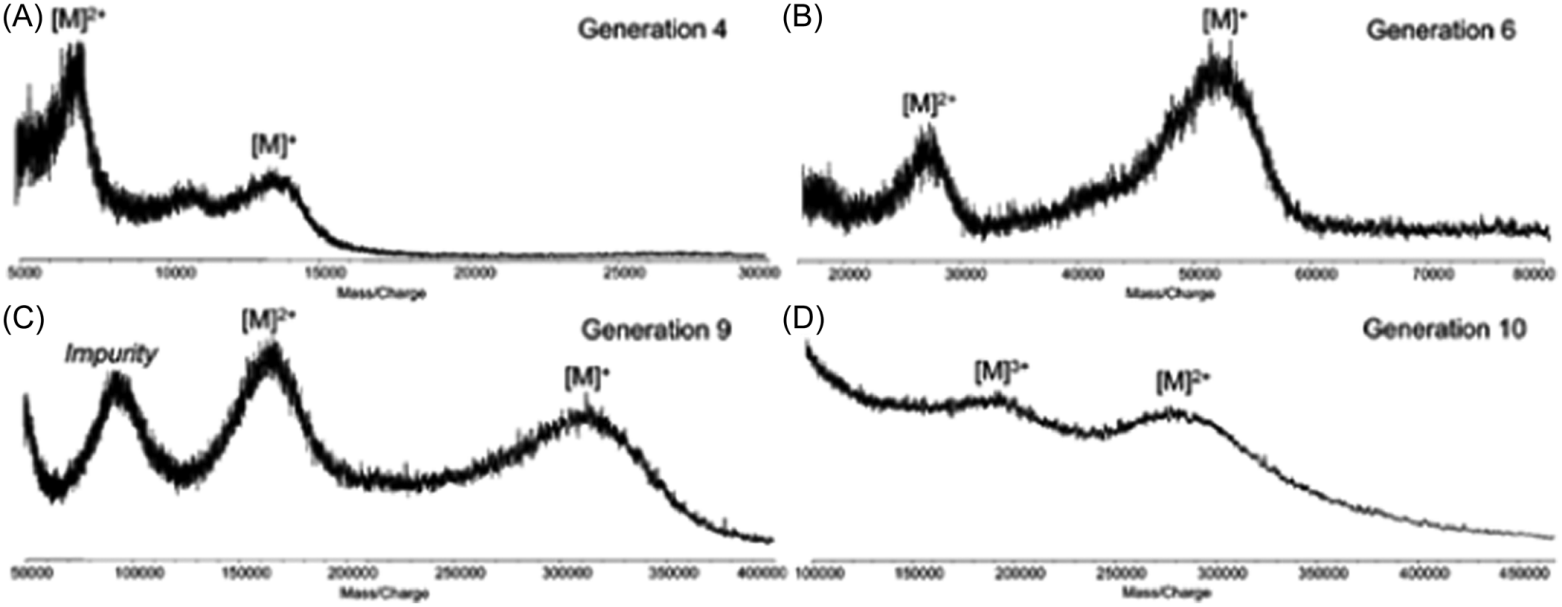
Positive mode matrix‐assisted laser desorption/ionization mass spectra of high generation of poly(amidoamine) (A) G4: m_th_ 14.21 kDa, m_exp_ 13.32 kDa; (B) G6: m_th_ 58.05 kDa, m_exp_ 50.16 kDa; (C) G9: m_th_ 467.14 kDa, m_exp_ 323.30 kDa; (D) G10: m_th_ 934.69 kDa, m_exp_ 579 kDa. Adapted from Muller et al. (2007), with permission from the American Chemical Society.

In MALDI, successful ionization of PAMAM was achieved with a large variety of matrices. The earliest publication from Zhou et al. (2001) reports MALDI of OH‐terminated PAMAM, using matrices such as 7‐HC for G2 (3.7 kDa) or THAP for G3 (6.9 kDa) and G4 (14.3 kDa), with no added salt (Zhou et al., 2001). Protonation of PAMAM G1 could be observed in MALDI using IAA (Shi et al., 2005) but Lopp and coworkers showed that proper choice of the matrix was mandatory for optimal production of protonated NH_2_‐terminated PAMAM while preventing their fragmentation (Peterson et al., 2003; Subbi et al., 2005). The softest matrix was found to be a mixture of 2,5‐DHB and fucose (1:1 molar ratio) but, as the generation grows, pure 2,5‐DHB was recommended to prevent increase of background noise assigned to trapping of sugar molecules inside the dendrimer framework. In contrast, fragmentation was observed near ionization threshold when using CHCA, consistent with the “hot” character of this matrix (Laugesen & Roepstorff, 2003). Interestingly, cationic adducts formed with ubiquitous Na^+^ or K^+^ showed much higher stability compared to protonated PAMAM (Subbi et al., 2005). Giordanengo et al. employed 2,5‐DHB to produce protonated molecules of fan‐shape NH_2_‐terminated PAMAM (G0–G1) but increase of the matrix‐to‐analyte ratio (from 1000:1 to 5000:1) together with addition of a sodium salt were required for successful MALDI of G2 and G3 as sodiated adducts (Giordanengo et al., 2007). Individual mass spectra of all components of a PAMAM G1 sample could be recorded by performing MALDI on a thin layer chromatography (TLC) plate used to separate the perfect dendrimer from its main impurities (Leriche et al., 2014). In this work, the TLC plate was dipped vertically in a 2,5‐DHB matrix solution after separation was achieved. Much larger NH_2_‐terminated PAMAM were investigated by the Allmaier group using an acidified solution of THAP to prepare MALDI samples (Muller & Allmaier, 2006; Muller et al., 2007). As illustrated in Figure 4, mass spectra obtained for these high‐mass species show PAMAM dendrimers at the +1 and +2 charge states, except for the largest G10 detected with 2 and 3 charges. Dimeric species were sometimes also observed. From these MALDI mass spectra recorded in the linear TOF mode, molecular weight of G2–G10 dendrimers was determined with a quite good precision (±0.4% on average) although using a mass spectrometer containing no special instrumental features optimized for detection of ultrahigh mass compounds. Yet, as detailed in the caption of Figure 4, molecular weights calculated from these MALDI data were systematically lower than masses expected for perfect dendrimers. The fact that these mass differences increased continuously with increasing generations was seen as a consequence of incomplete synthesis of PAMAM.

For dendrimers like PAMAMs prepared by divergent process, different strategies were reported for safe assignment of additional signals to defective dendrimers rather than to ionic fragments generated during the MALDI process. Unlike fragments,

– abundance of defects does not increase with the laser power (Zhou et al., 2001);
– these impurities are also observed using an alternative ionization technique such as ESI (Giordanengo et al., 2007; Shi et al., 2005);
– they can be separated from the perfect dendrimer based on their ion mobility (Muller et al., 2007) or distributed in different fractions collected after chromatographic purification (Peterson et al., 2003);
– last but not least, dendritic defects are most often detected at different *m/z* values compared to fragments obtained during post‐source decay (PSD) experiments (Subbi et al., 2005).

Due to propagation of these impurities from one generation to the other, the complexity of MALDI mass spectra increases rapidly with PAMAM dendrimer size. Yet, data interpretation could be simplified using models enabling prediction of molecular mass of structural deviations depending on the reactivity of defective molecules from one generation to the following one (Giordanengo et al., 2007).

#### 2.1.3 Polypropyleneimine (PPI) dendrimers

PPI dendrimers (also named polypropyleneamine, POPAM) are synthesized via a divergent approach, starting from diaminobutane (DAB) and alternating sequence of Michael reaction (yielding nitrile end‐groups) and hydrogenation reaction (to form amine terminations). Both DAB‐dendr‐(CN)_*x*_ and DAB‐dendr‐(NH_2_)_*x*_ are very polar and polybasic compounds, which makes them well suited for ESI, as first reported by Meijer and coworkers who analyzed G2–G5 PPI dendrimers solubilized in water/methanol (75/25, v/v) (Hummelen et al., 1997). Deconvolution of multiply charged species (from +4 to +12) revealed the perfect molecules as well as impurities expected from the divergent synthesis of PPI. On the one hand, incomplete Michael addition leads to a mass defect of 53 Da (missing acrylonitrile) in DAB‐dendr‐(CN)_*x*_, further leading to a 57 Da mass defect (missing propylamine) when considering DAB‐dendr‐(NH_2_)_*x*_. On the other hand, cyclization connecting two branches at their ends through the formation of a secondary amine can occur during the hydrogenation step and is revealed by species with a 17 Da mass defect. Assuming similar ionization yields for perfect and imperfect dendrimers, the authors further derived the probability of these two side reactions from ESI mass spectra of low generations and were able to successfully simulate spectra of high generation. Performing chromatographic separation before ESI permitted to evidence multiple isomers of imperfect PPI dendrimers but at the cost of analysis time and with no precise information regarding location of defective moieties (van der Wal et al., 1998). Overestimation of some PPI defects in ESI‐MS has been reported by the Schalley group who found that MALDI data were more representative of PPI dendrimers, using the same CHCl_3_/MeOH (3/1, v/v) binary mixture to dissolve the DHB matrix and the G1 DAB‐dendr‐(NH_2_)_4_ (Baytekin et al., 2006). Using a different matrix (DIT), Wesdemiotis and coworkers recommended the use of a ternary solvent mixture, namely acetonitrile/ethanol/water (50:45:5, v/v/v), for MALDI sample preparation: as compared to any other pure or binary solutions of methanol, ethanol and water, this ternary mixture enabled substantially higher signal‐to‐noise ratio for the G2 DAB‐dendr‐(NH_2_)_16_ (Adhiya & Wesdemiotis, 2002). The sole issue reported so far during MALDI of PPI was related to small DAB‐dendrimers with neither CN nor NH_2_ terminations: protonation of core tertiary amines was followed by dehydrogenation, yielding [M – H]^+^ ions instead of the expected [M + H]^+^ species (Lou et al., 2008). This phenomenon was observed to be matrix‐dependent: loss of H_2_ was favored when using “hot” matrices such as CHCA or SA while it could be minimized with DHB.

#### 2.1.4 Phosphorus dendrimers

**Figure 5:**
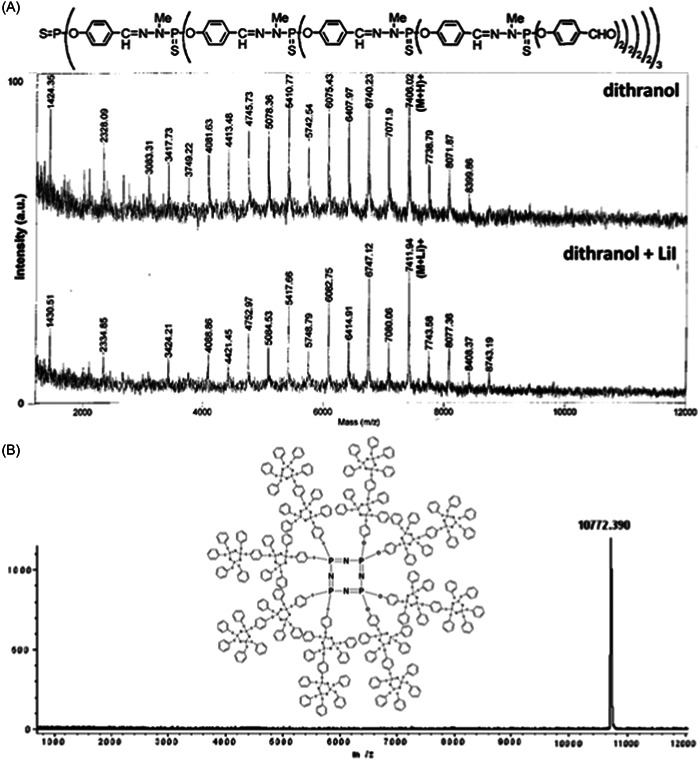
(A) Positive mode matrix‐assisted laser desorption/ionization (MALDI) mass spectrum of phenoxymethyl(methylhydrazono)) dendrimer G3 using the dithranol matrix without (top) or with (bottom) lithium iodide. Adapted from Blais et al. (2000), with permission from the American Chemical Society. (B) Positive mode MALDI mass spectrum of cyclic phosphazene dendrimer using the 1,8,9‐ACT matrix. Adapted from Cosut et al. (2011), with permission from Elsevier B. V.

Since ^31^P NMR is very sensitive to small changes in nuclei environment, it remains by far the method of choice to characterize phosphorus‐containing dendrimers (Caminade et al., 2010). Consequently, only two articles were found with detailed MALDI‐MS analysis of phosphorus dendrimers, although with different outputs. The first study dealing with poly(thiophosphoryl phenoxymethyl(methylhydrazono)) or PMMH dendrimers reports dissociation of the targeted species upon laser irradiation (Blais et al., 2000). The extent of dissociation increases with the generation number (from G1 to G4), consistent with a red shift of the broad absorption spectra for the highest generations. Fragmentation could be slightly limited by using DIT (which required a lower laser fluence compared to 2,5‐DHB) and lithium adduction instead of protonation as the ionization mode. Beside signals of intact dendrimers, MALDI mass spectra showed signals indicative of both the loss and gain of mass (Figure 5A), which could be rationalized by considering intramolecular and intermolecular reactions. Such an extensive degradation was obviously not observed for phosphorus dendrimers that do not absorb the 337 nm wavelength of UV laser. As exemplified in Figure 5B, very clean MALDI mass spectra with intense protonated molecules were recorded for phosphazene‐based dendrimers using 1,8,9‐ACT as the matrix (Cosut et al., 2011).

#### 2.1.5 Carbosilane dendrimers

**Figure 6:**
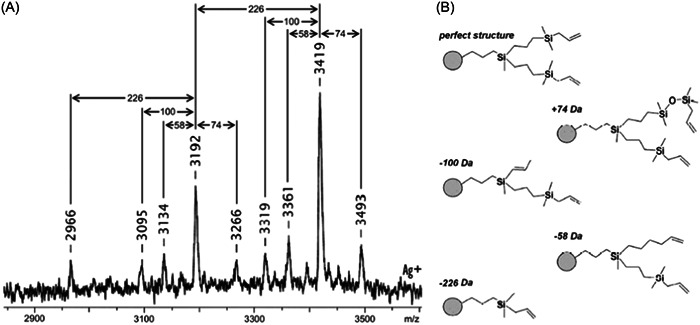
(A) Positive mode matrix‐assisted laser desorption/ionization mass spectrum of polyallylcarbosilane (G3‐allyl_16_) dendrimer (*m/z* 3419) obtained with 1,8,9‐ACT supplemented with silver trifluoroacetate and (b) assignment of defective branches based on mass difference from the perfect structure. Adapted from Krupkova et al. (2010) with permission from the American Chemical Society.

Carbosilane‐based dendrimers have silicon atoms at the center and at the branching points and, although generally characterized by ^29^Si NMR (Lambert et al., 1998), they were also studied by MS. Their first MALDI analysis was reported in 1995, from G0 (4 end‐groups) to G3 (108 end‐groups), with different experimental conditions depending on their terminations: for dendritic polyols, MALDI was performed with 2,5‐DHB supplemented with sodium acetate whereas 5‐CSA with silver trifluoroacetate was found best for their allyl‐terminated precursors (Lorenz et al., 1995). The same two matrices were employed by Wu and Biemann when investigating the MALDI behavior of carbosilane dendrimers as a function of their termination polarity (Wu & Biemann, 1997). This latter work also demonstrates that experimental conditions had to be adapted to the nature of end‐groups. When bearing basic terminations such as trimethylamine moieties, carbosilane dendrimers were observed to readily protonate using 2,5‐DHB as the matrix. In contrast, combination of a silver salt with 5‐CSA was requested for dendrimers terminated by non‐polar chloroalkyl groups to be ionized as Ag^+^ adducts. In the negative ion mode, successful deprotonation of carbosilane dendrimers holding sodium alkylsulfonate terminations could only be achieved after exchange of Na^+^ counter‐ions by NH_4_^+^, using 2,5‐DHB as the matrix. Interestingly, these MALDI mass spectra always exhibited singly charged [M – H]^–^ ions regardless of the total number of end‐groups in the dendrimer. Similarly, for dendrimers having fixed positive charges in their trimethylammonium chloride (or iodide) terminations, species were always detected as mono‐cations obtained after removal of one halide and exchange of all other ones by carboxylate anions from the matrix. Yet, these complexes were only observed to form with either 2,5‐DHB or 5‐CSA (i.e., two structural analogues) whereas MALDI experiments performed with alternative matrices such as CHCA or SA failed. Carbosilane dendrimer samples may also contain synthesis by‐products since they are typically prepared via the divergent approach, growing the molecule outward from the core by alternation of hydrosilylation of double bonds with chlorosilanes and nucleophilic substitution of chlorine with alkenylmagnesium halides (Frey & Schlenk, 2000). As detailed by Krupkova et al., most defects are actually formed during the hydrosilylation step and, since products of this reaction cannot be purified due to their high moisture sensitivity, defects rapidly propagate as the dendrimer generation grows (Krupkova et al., 2010). This is illustrated in Figure 6 with structural assignment proposed for all species detected in the MALDI mass spectrum of a polyallylcarbosilane dendrimer, using a predictive model for defect probability (Krupkova et al., 2007).

#### 2.1.6 Polysulfonamide dendrimers

**Figure 7:**
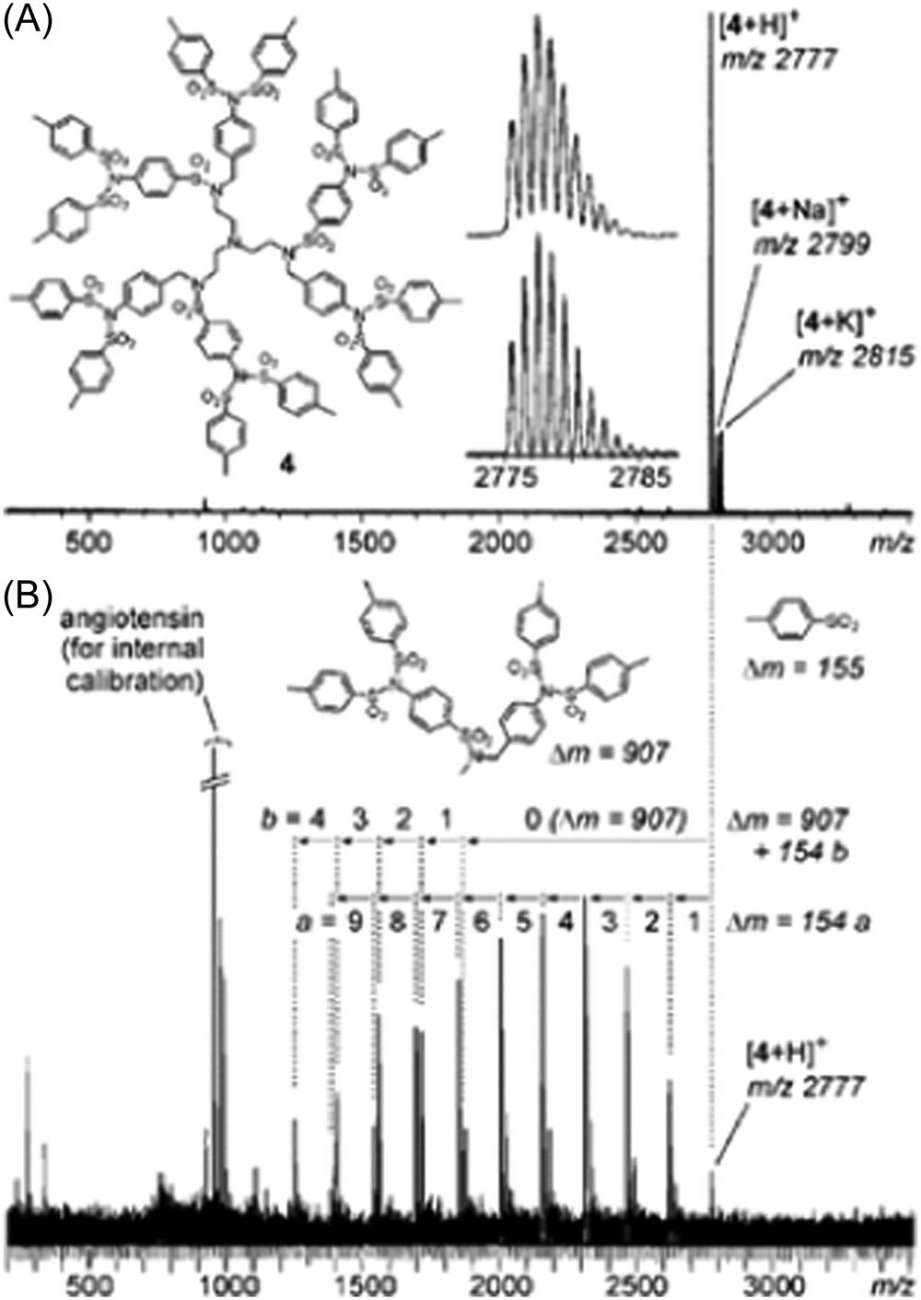
Positive mode mass spectra obtained after (A) electrospray ionization and (B) matrix‐assisted laser desorption/ionization of oligosulfonamide dendrimer (structure shown in inset) using 2,5‐DHB as the matrix. Reprinted from Felder et al. (2005), with permission from Wiley‐VCH Verlag GmbH & Co.

Dendrimers with aromatic sulfonamide groups at each branching point have unique properties such as high melting and glass transition temperatures, permitting applications in quite harsh environments (Bondareva et al., 2020). However, in spite of their high chemical stability, these dendrimers were reported to react with acidic matrices, leading to false negative results in MALDI‐MS (Felder et al., 2005). While mass data recorded after ESI were indicative of a sample with quite high monodispersity (Figure 7A), the protonated dendrimer was hardly detected in the MALDI mass spectrum obtained with 2,5‐DHB which, instead, showed two series of intense signals spaced by 154 Da (Figure 7B). Nucleophilic attack of 2,5‐DHB onto a protonated sulfonamide group was proposed to explain successive losses of branches as sulfoxides, leading to the first ion series starting from the protonated dendrimer and composed of up to nine congeners. In contrast, the second ion series formed after the loss of the whole 907 Da branch would originate from unimolecular dissociation in the gas phase. These assignments were clearly supported by MS/MS experiments: upon collisional activation of the protonated dendrimer, the first series was not observed, hence confirming that these ions result from reactive MALDI, whereas formation of the second series was a preferential fragmentation pathway (see Section 2.2.3). Lowering the acidic character of the matrix by changing 2,5‐DHB to 9‐NA or DCTB permitted to avoid the reactive MALDI phenomenon but did not prevent the 907 Da loss leading to the second ion series, making ESI the preferential technique to qualify the purity of such dendrimer samples (Lukin et al., 2006). Promoting formation of alkali adducts by adding a small amount of sodium chloride seemed to avoid these issues, as reported by Schubert et al. who used a 700‐fold excess of the DIT matrix to perform MALDI of small sulfonamide dendrimers (Schubert et al., 2008).

#### 2.1.7 Poly(aminoester) dendrimers

**Figure 8:**
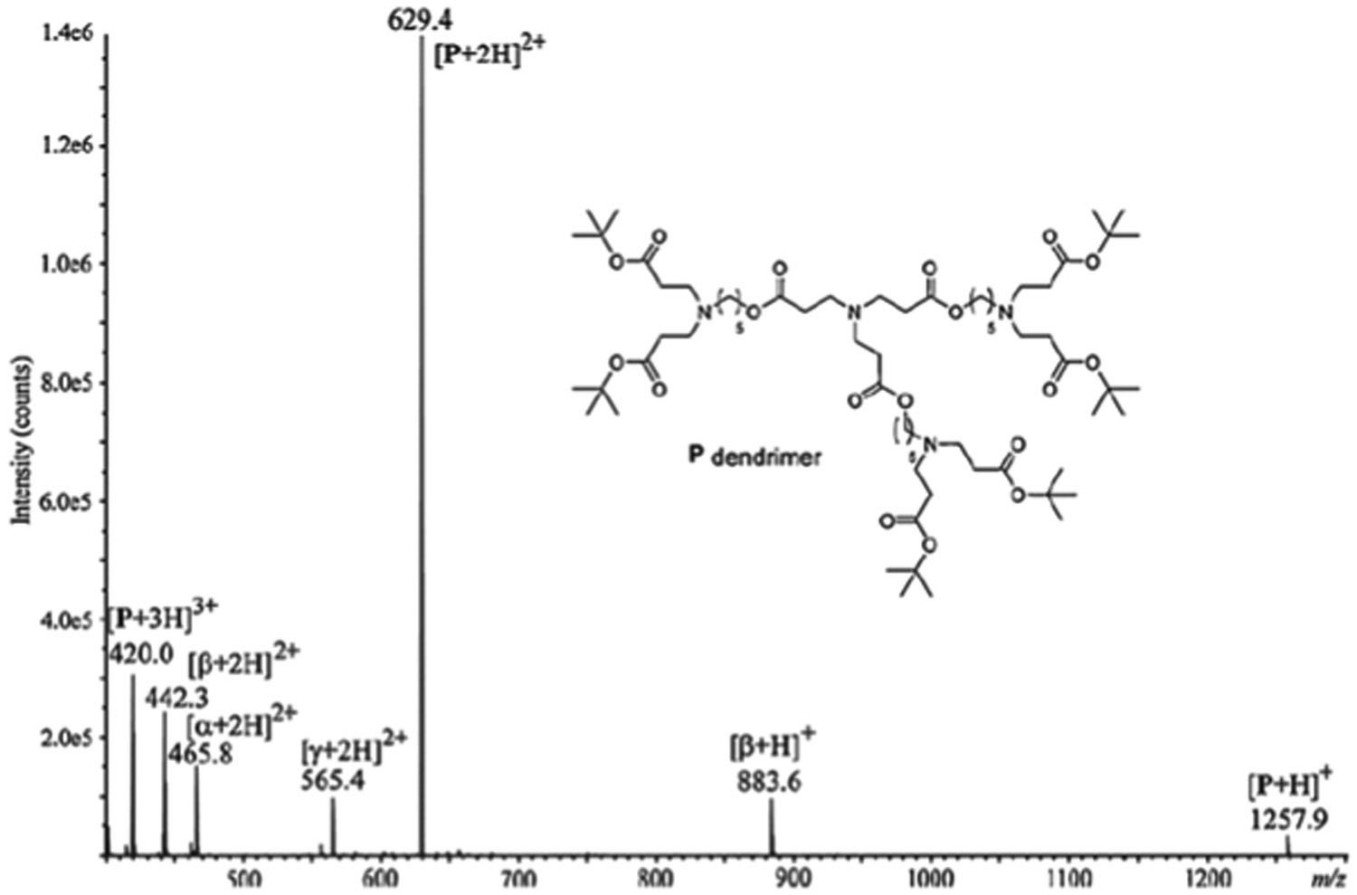
Positive mode electrospray ionization mass spectrum of poly(aminoester) dendrimer (named P, structure shown in inset, 1256.9 Da) observed as [P + *n*H]^*n*+^ (*n* = 1–3) together with three minor impurities named α (929.6 Da), β (882.6 Da), and γ (1128.8 Da). Reprinted from Tintaru et al. (2011), with permission from Elsevier B. V.

Dendrimers containing both ester and amine functions were designed to combine biodegradability properties (via ester hydrolysis) with encapsulation abilities offered by amine groups, two appealing advantages for drug delivery applications (Quelever et al., 2013). Presence of these two functionalities makes their divergent synthesis quite difficult (Bouillon et al., 2010) and minor impurities were indeed reported in ESI‐MS of poly(aminoester) dendrimers, also named poly(esteramine) dendrimers (Tintaru et al., 2010, 2011). As documented in Figure 8, most abundant ions were protonated molecules (+1 to +3 charge states) of the targeted dendrimer but three other species were also clearly detected. Amongst them, only the impurity named γ could be intuitively assigned based on its 129 Da mass defect corresponding to the mass of one branch. Yet, performing MS/MS experiments enabled precise characterization of the unknowns α and β impurities, as detailed in Section 2.2.4.

#### 2.1.8 Polyester dendrimers

**Figure 9:**
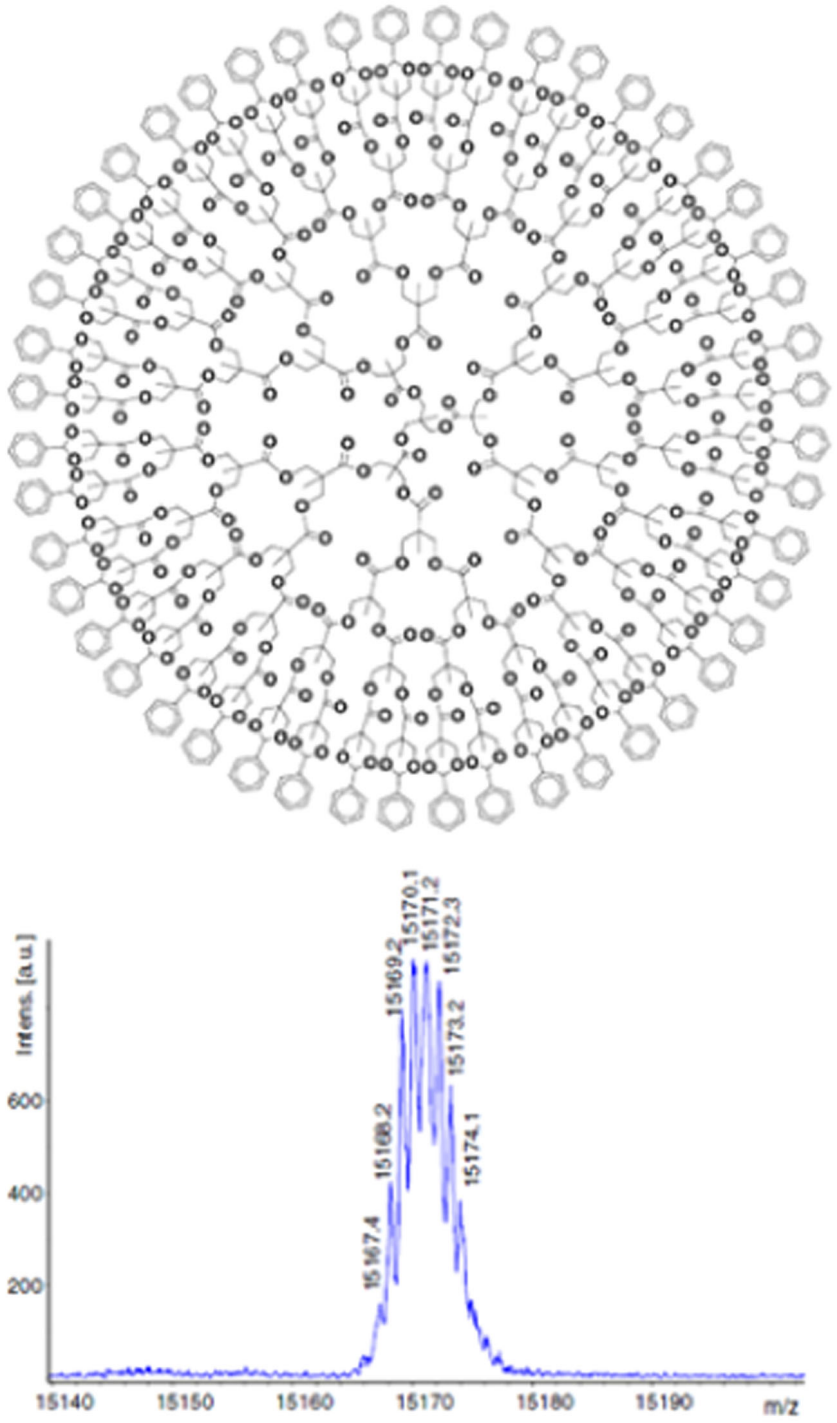
Matrix‐assisted laser desorption/ionization mass spectrum of bis‐MPA‐based dendrimer with the calculated average mass of 15,172.19 Da (top) using 9‐NA as the matrix and NaTFA as the cationing agent. Reprinted from Grayson et al. (2014), with permission from the American Chemical Society. [Color figure can be viewed at wileyonlinelibrary.com]

Successful MALDI production of aromatic polyester dendrimers was first reported by Haddleton et al., with molecules ionized as sodium adducts from G1 to G3 generations (Sahota et al., 1994). A layered sample preparation was employed, first depositing an aliquot of the 2,5‐DHB matrix solution then a droplet of the dendrimer solution doped with NaCl. Similar quality data were obtained using the same matrix regardless of dendrimer terminations (benzyl ether vs. hydroxyl groups) and the same matrix/analyte molar ratio independently of the dendrimer size, as observed up to molecular weight of about 5200 Da. Low laser fluence is however recommended for MALDI of such aryl ester dendrimers prone to laser‐induced fragmentation (Clark et al., 1999). Beside 2,5‐DHB, MALDI of aromatic polyester dendrimers was also achieved with a variety of other matrices such as SA and CHCA (Haddleton et al., 1996) as well as DIT and 9‐NA (Mowat et al., 1998). Later on, the exceptional tolerance of polyester dendrimers towards experimental conditions was demonstrated through their compatibility with an extended selection of matrices (Grayson et al., 2014). In this study, sodium adducts of bis(hydroxymethyl)propanoic acid (bis‐MPA)‐based dendrimers were successfully generated by MALDI with all tested 14 matrices, as exemplified in Figure 9 with 9‐NA, but also by surface‐assisted laser desorption/ionization (SALDI) with either graphite or silicon nanowires. Using 9‐NA, MALDI mass data of similar quality could also be obtained from dendrimer samples prepared in a wide variety of solvents, ranging from apolar (e.g., hexane) to polar (e.g., methanol) media, the later conditions making these polyester dendrimers also amenable to ESI (Romson et al., 2021). This high tolerance to a large variety of experimental conditions was one of the key criteria to select these (bis‐MPA)‐based dendrimers as ideal standards for mass calibration in MALDI (see Section 3.1).

#### 2.1.9 Polyether dendrimers

**Figure 10:**
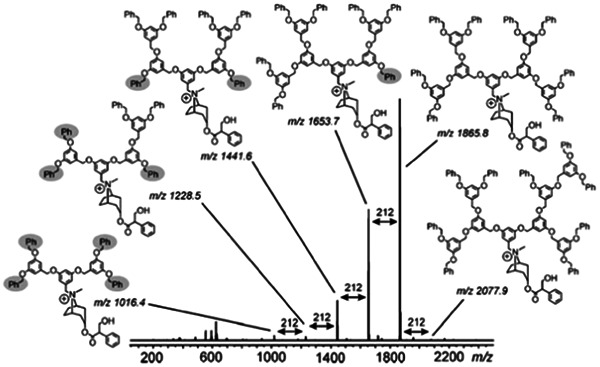
Electrospray ionization Fourier transform ion cyclotron resonance mass spectrum of the third generation Fréchet dendron after conversion of the benzyl bromide focal point into quaternary ammonium cation. Reprinted from Baytekin et al. (2009), with permission from Wiley‐VCH Verlag GmbH & Co.

The vast majority of MS studies found for this dendrimer category deals with poly(aryl ether) species, commonly prepared according to convergent synthetic processes. Accordingly, reported works applied MS to characterize either dendrons or full dendrimers. The earliest work was reported in 1995, with MALDI of polyether dendrimers of different molecular weights successfully achieved using IAA as the matrix, regardless of their terminal functionalities, ester versus perdeuterophenyl groups (Leon & Frechet, 1995). Other tested matrices such as SA, 3,5‐DHB, and CHCA failed at producing MS signals. Although no salt was added to the IAA/sample mixtures, dendrimers were readily desorbed as alkali adducts such as [M + Na]^+^ and/or [M + K]^+^. In contrast, addition of small amounts of LiCl or sodium dodecyl sulfate seemed to affect crystallization during sample drying due to the poor solubility of these salts in THF, the preferred solvent for these dendrimers. The matrix/analyte ratio was optimized as a function of molecular weight, with lower matrix/analyte ratio observed to be more beneficial to low‐mass dendrimers (<3000 Da). The same group later reported extensive clustering during MALDI of acidic‐terminated poly(aryl ether) dendrimers, with aggregates containing up to nine molecules (Leon et al., 1996). Zhao et al. have investigated the influence of cationizing agents in MALDI of Fréchet‐type dendrons (Zhao et al., 2003) and found that, in spite of their low molecular weight (<2000 Da), cationization efficiency was enhanced by addition of cesium whereas it was very poor with lithium. These results were rationalized by molecular modeling showing that, compared to Li^+^, the larger Cs^+^ cation was best solvated by two or three ether oxygen atoms, hence yielding a more stable ionic adduct. In contrast, the more flexible structure of aryl ether‐like polypentylresorcinol dendrimers was shown to readily accommodate any alkali when using CHCA as the matrix (Neubert et al., 2003). When examining polyether dendrimers with a pyridine‐based skeleton, Traldi and coll. found that cationized species could readily form by laser desorption/ionization (LDI) whereas the use of a matrix led to extensive fragmentation during MALDI (Chessa et al., 1998). The extent of dendrimer dissociation in the gas phase could be limited by changing 2,5‐DHB to CHCA or DIT but could not be rationalized based on relative proton affinity of these matrices. ESI‐MS analysis of Fréchet‐type dendrons was also reported, using different experimental conditions as a function of the chemical groups at their focal point (Baytekin et al., 2009). The negative ion mode favored deprotonation for species holding a benzylic hydroxy group whereas their benzyl bromide counterparts had first to be converted to quaternary ammonium cation before positive mode ESI (Baytekin et al., 2009). The latter conditions enabled high signal‐to‐noise ratio for best accurate mass measurement of G1–G2 generation dendrons using FT‐ICR‐MS and permitted to highlight the presence of defective molecules produced during preparation of the G3 sample. As documented in Figure 10, these species appear at mass distances of 212 Da below (and to a lesser extent above) the perfect structure at *m/z* 1865.8 and reveal rearrangements of the benzyl ether linkages due to acid traces cogenerated during the conversion of the benzyl alcohol into benzyl bromide with PBr_3_. This side reaction could be avoided by the alternative use of CBr_4_/Ph_3_P to produce the benzyl bromide at the focal point.

### 2.2 Structural characterization by MS/MS

As shown throughout the previous section, assignment of additional signals to particular dendrimer impurities in spectra recorded in the MS mode is commonly done based on mass information only, since synthesis side‐products usually differ from ideal expected structures by a known amount of mass. Yet, although it can be considered as primary evidence, mass does not provide structural information. In contrast, MS/MS remains the cornerstone in any work related to molecular characterization because it produces fragments that allow insights in the connectivity of functional groups in the dissociating precursor. Understanding gas‐phase chemistry of dendrimers is hence a primary requirement to build guidelines on how to deduce structural information from fragments observed in MS/MS. Once established for the perfect dendrimer, dissociation rules can then be usefully employed to deduce the structure of defective species because any deviation from specific fragmentation pathways is the result of a flawed section of the structure. This approach is discussed hereafter based on the detailed fragmentation studies reported for five main dendrimer classes.

#### 2.2.1 MS/MS of PAMAM dendrimers

**Figure 11:**
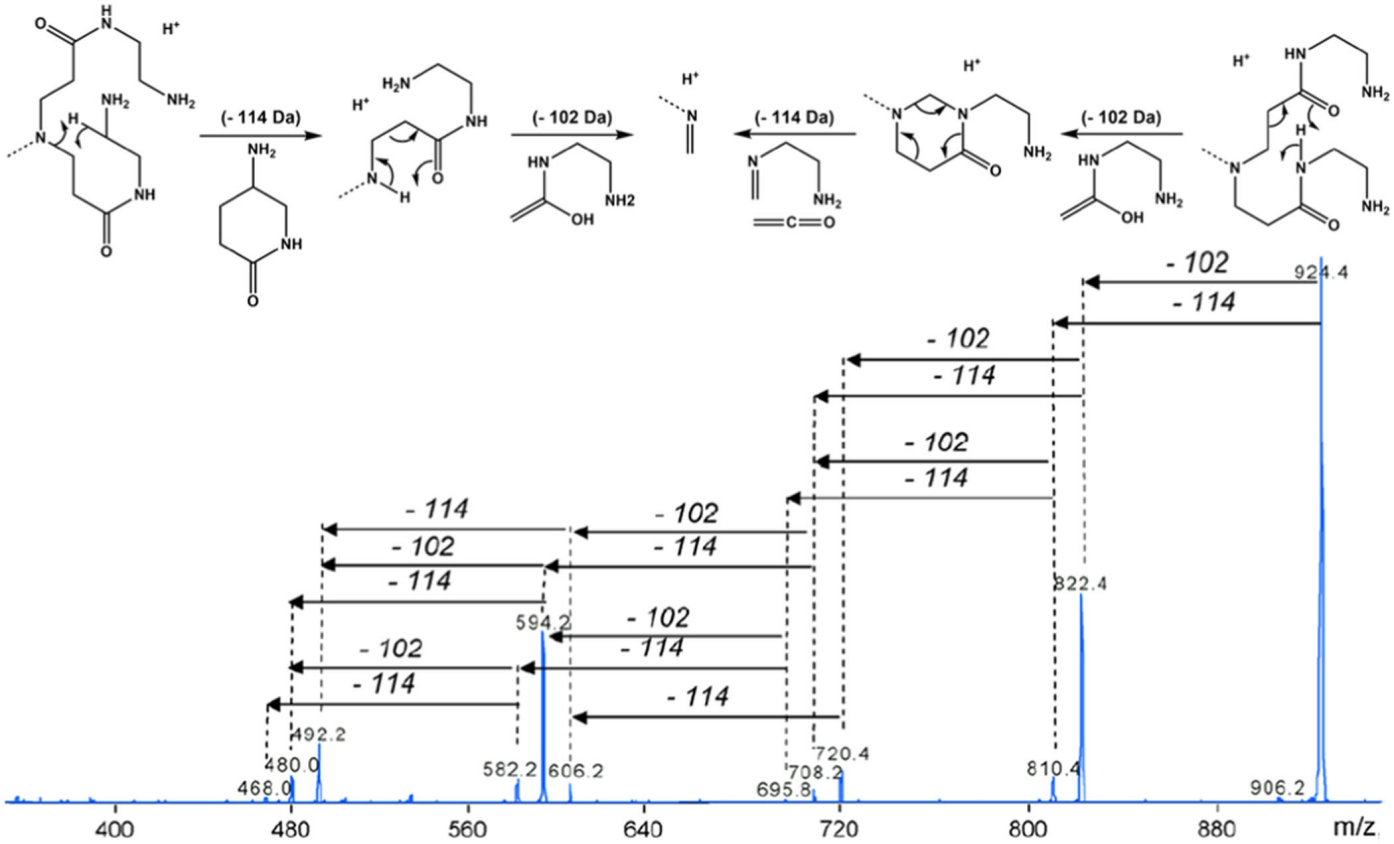
Collision‐induced dissociation spectrum of protonated fan‐shape polyamidoamine G1 at *m/z* 924.4. Top: mechanisms proposed to account for elimination of 114 Da then 102 Da (left part) or for release of 102 Da before 114 Da (right part). Adapted from Giordanengo et al. (2007), with permission from Elsevier B. V. [Color figure can be viewed at wileyonlinelibrary.com]

**Figure 12:**
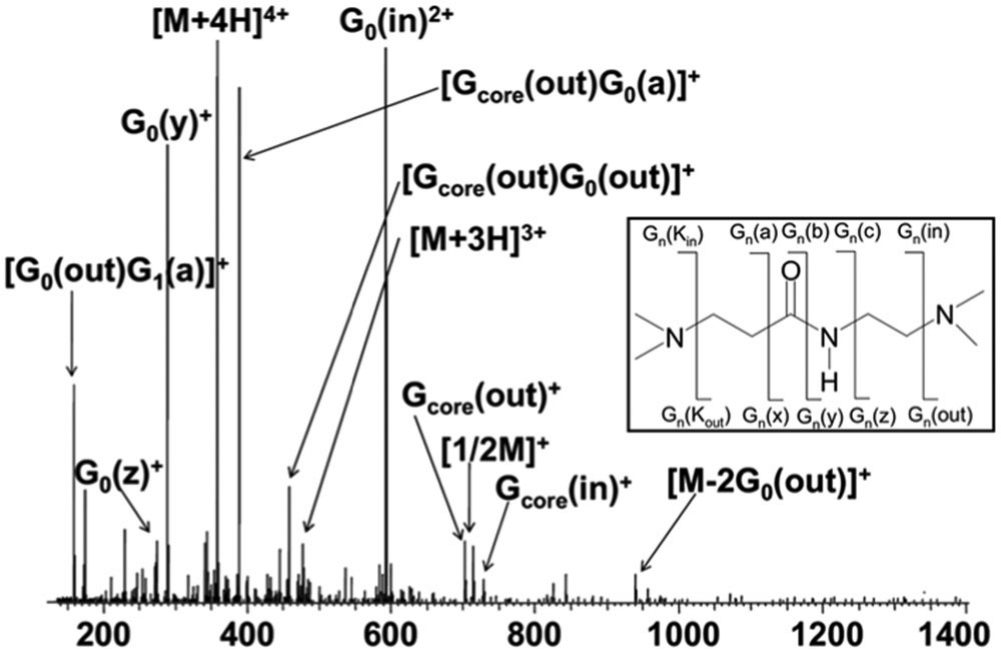
Electron capture dissociation (ECD) Fourier‐transform ion cyclotron resonance spectrum of [PAMAM(G1)‐NH_2_ + 4H]^4+^, with notations for ECD cleavage sites along the backbone of PAMAM dendrimer. Adapted from Kaczorowska and Cooper (2008), with permission from the American Chemical Society.

Gas‐phase unimolecular dissociation of PAMAMs has been investigated using a wide variety of activation methods, which detailed principle and mode of operation can be found in dedicated tutorial reviews (Bayat et al., 2020; Sleno & Volmer, 2004). Fragmentation of full generation NH_2_‐terminated PAMAMs was first investigated by performing collision‐induced dissociation (CID), using ESI to generate protonated species (Giordanengo et al., 2007). Two main dissociation pathways were identified, consisting of losses of 114 and 102 Da neutrals observed to occur either competitively or consecutively from terminal branches of the dendrimer (Figure 11). With support of hydrogen/deuterium exchange (HDX) experiments, different mechanisms could be proposed depending on the 114 Da loss proceeding before or after elimination of the 102 Da neutral (Figure 11, top scheme). Elimination of a 114 Da branch can also be seen as a retro‐Michael reaction when considering that it proceeds via 1,2‐proton transfer. Occurrence of these two reactions was later evidenced in MS^*n*^ experiments performed in an ion trap (Vincent et al., 2008). Such a fragmentation pattern was useful and helped confirming the structure of impurities detected in PAMAM samples, since the number of 114 Da/102 Da neutral losses is obviously lowered in case of “molecular loop” or “missing arm” defects (Giordanengo et al., 2007). When increasing the precursor ion charge state, the same fragmentation pattern was observed but loss of water (observed to a minor extent in Figure 11) became the dominant reaction. This dehydration process was proposed to occur upon protonation of a terminal primary amine, ultimately yielding a five‐membered aminopyrroline ring (Kaczorowska & Cooper, 2008). Additional reactions were observed during CID of cationic adducts of NH_2_‐terminated PAMAM. For silver adducts, the same reaction leading to the release of one branch was observed to proceed at the dendrimer core, hence causing elimination of one arm (–342 Da). Ag^+^ adduction also induced bond cleavage between the methylene and carbonyl bond, as revealed by release of 86 Da neutral (Mazzitelli & Brodbelt, 2006). Beside the 114 Da/102 Da sequential losses, sodiated PAMAM experienced ethylene diamine (60 Da) elimination indicative of the amide bond cleavage (Ulaszewska et al., 2013; Vincent et al., 2008). The latter reaction, also observed from lithiated PAMAM, was particularly useful to localize the modified moiety in the “+12 *m/z*” defective molecules and assess their structure (Tintaru et al., 2015). In contrast to CID, electron capture dissociation (ECD) (Zubarev et al., 1998) of multiply protonated NH_2_‐terminated PAMAM was shown to dominantly proceed by bond cleavage in the interior of the dendrimer (Kaczorowska & Cooper, 2008). Indeed, all major ECD products originate from the inner G0 layer as indicated by the nomenclature defined by Ho and coworkers (Lee et al., 2006) used to designate fragments as G_*n*_(m) in Figure 12, where *n* refers to the generation layer in which fragmentation takes place and m describes the type of fragmentation.

CID of half‐generation PAMAMs, most often available as sodium salts and best ionized in the negative ion mode thanks to their carboxylate end‐groups, was reported to mainly proceed via retro‐Michael rearrangements (He & McLuckey, 2004b). As demonstrated for multiply charged precursors generated during ESI, such a reaction induced cleavage of one branch, either released as a 72 Da neutral or detected as a *m/z* 71 deprotonated species when occurring at one of the most exterior tertiary nitrogens. Similarly, release of one arm was revealed by loss of 280 Da and detection of the *m/z* 279 anion when the cleaved bond was at one of the interior tertiary nitrogens. Based on these dissociation rules, an in‐depth fragmentation study employing MS^*n*^ experiments permitted to identify a PAMAM impurity containing a branch extension (He & McLuckey, 2004b). Dissociation was also studied for half‐generation PAMAMs containing some proton counter‐ions after H/Na exchange in some end‐groups: interestingly, elimination of the entire arm was no longer a competitive process in CID (He & McLuckey, 2004b) whereas, when performing electron detachment dissociation (EDD) (Budnik et al., 2001), this useful reaction was still clearly observed (Kaczorowska & Cooper, 2008).

Due to their commercial availability, PAMAMs with amidoethanol terminations were also studied with different activation techniques. CID of these protonated dendrimers was shown to consist of combined losses of two neutrals in various stoichiometries, a 103 Da molecule corresponding to CH_2_ = C(OH)‐NHCH_2_CH_2_OH formed after C–C bond cleavage and the 115 Da branch detached upon cleavage at the tertiary amine (Kaczorowska & Cooper, 2008). The influence of different metal adduction on the CID behavior of these dendrimers was also investigated in this study. While fragmentation of precursor ions such as [M + Ag + 4H]^5+^, [M + Zn + 3H]^5+^, and [M + Fe^II^ + 3H]^5+^ mostly proceeded similar to fully protonated dendrimers at the same 5+ charge state, adduction of Cu^2+^ or Fe^3+^ drastically modified the CID pattern, with the main C–C bond cleavage in the EDA core suggesting coordination of these cations by the two core nitrogen atoms (Kaczorowska & Cooper, 2009). As previously shown for NH_2_‐terminated PAMAM, dominant fragmentation channels observed during ECD of PAMAM with amidoethanol end‐groups were cleavage at the tertiary amine and pronounced amide bond cleavage, mostly occurring in the innermost generation layers (Kaczorowska & Cooper, 2008; Lee et al., 2006). Similar radical‐induced pathways were observed during the in‐source decay of MALDI ions generated from these dendrimers (So et al., 2012). Electron capture by the metal upon ECD of dendrimers adducted with Cu^2+^ or Fe^3+^ yielded charge‐reduced complexes with prevalent cleavages core‐side of the amide bond (Kaczorowska & Cooper, 2009). In summary, as compared to CID of PAMAM observed to largely depend on the nature of the surface groups, electron‐mediated techniques produce more simple spectra containing fragments from bond cleavage in the innermost generation layers and are hence appealing for structural characterization of large PAMAMs.

#### 2.2.2 MS/MS of PPI dendrimers

**Figure 13:**
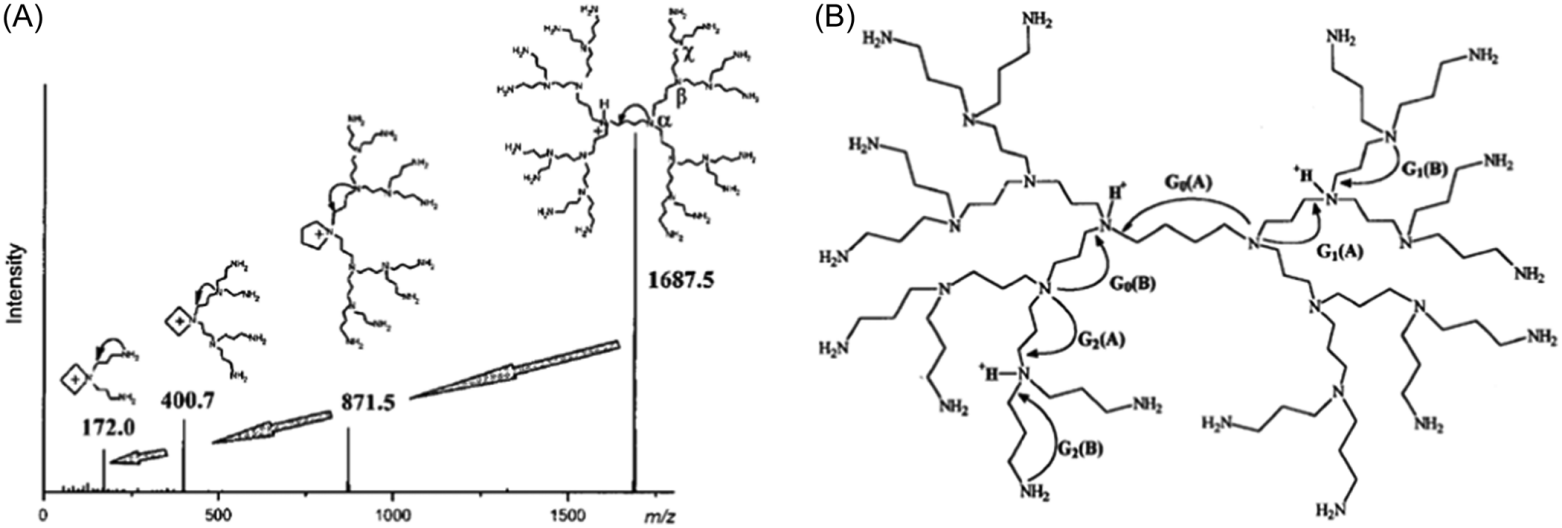
(A) Main collision‐induced dissociation pattern of singly protonated polypropyleneimine (PPI) G3 (also named DAB‐dendr‐(NH_2_)_16_) described to proceed via consecutive SNi reactions. Reprinted from Weener et al. (1999), with permission from the American Chemical Society. (B) Alternative competitive dissociation channels as a function of the protonated tertiary nitrogen atom in PPI G3. Reprinted from McLuckey et al. (2000), with permission from Elsevier B. V.

Fragmentation of DAB‐core PPI (G1–G5) with NH_2_ terminations has been thoroughly investigated by CID. Occurrence of intramolecular nucleophilic substitution (SNi) reactions was demonstrated, yielding different dominant fragments as a function of the precursor ion charge state. With the proton located on one of the DAB nitrogen atoms, singly charged PPIs would first cleave at their central core, leading to a charged heterocyclic ring which acts as the leaving group in the subsequent nucleophilic attack, as illustrated by the reaction cascade shown in Figure 13A (Weener et al., 1999). All fragments observed in this MS/MS spectrum could also be generated directly from the protonated precursor when considering protonation of alternative ternary amines and competing SNi reactions (de Maaijer‐Gielbert et al., 1999; McLuckey et al., 2000). As depicted in Figure 13B, attack of the carbon alpha to the charged site can occur from the adjacent nitrogen closer to the interior (process A) or closer to the exterior (process B) of the dendrimer. In other words, a direct correlation can be established between the fragmentation sites and the protonated sites. Accordingly, the *m/z* 871.5 fragment observed in Figure 13A would result from the inside‐to‐outside attack named G_0_(A) whereas outside‐to‐inside attacks respectively labelled G_0_(B) and G_1_(B) would account for the formation of *m/z* 400.7 and *m/z* 172.0 fragments, respectively. Taking advantage of the fact that products of the major G_0_(A) and G_0_(B) processes respectively contain roughly one‐half and one‐quarter of the dissociating singly charged PPI, McLuckey et al. could obtain compositional information about isomeric mixtures of PPI defects: it was found that repeated side‐reactions such as multiple failed Michael additions (leading to missing propylene imines of mass 57 Da) tend to occur on one side of the dendrimer (McLuckey et al., 2000). By monitoring the G_0_(A) pathway mainly experienced by PPI‐based persulfonated dendrimers despite aromatic sulfonamide groups were present at each branching point, Felder et al. were able to identify all defects found in the G1 sample (Felder et al., 2005). In contrast, Adhiya and Wesdemiotis focused on the minor G_1_(A) and G_2_(A) fragmentation channels to highlight conformational differences of protonated DAB‐dendr‐(NH_2_)_16_ as a function of the solvent used for their MALDI sample preparation (Adhiya & Wesdemiotis, 2002). As compared to polar protic solvents, use of benzene was observed to prevent elimination of outer branches of mass 131.1 Da according to G_2_(A) or 359.4 Da via G_1_(A), as evidenced in PSD experiments. As a poor PPI solvent, benzene would favor a folded conformation where the peripheral branches point into the interior to enable intramolecular self‐solvation via hydrogen bonding, hence preventing some C–N bonds to be efficiently cleaved. The authors noted that the same conformational differences were detected in liquid state NMR as a function of the solvent (polar protic vs. nonpolar) used to dissolve PPIs (Chai et al., 2001), strongly suggesting that the dendrimer structure prevalent in solution was maintained in the gas phase.

For highly charged precursors, main dissociation reactions are directed by protonation closer to the periphery of the PPI dendrimer, with major fragments being the *m/z* 172 quaternary ammonium ion and its complementary product (e.g., pathway G_1_(B) in Figure 13B) as well as the ion obtained after elimination of the 131 Da amine (e.g., pathway G_2_(A) in Figure 13B). This was observed for PPIs electrosprayed as multiply charged species and subjected to CID in triple quadrupole mass spectrometer (Weener et al., 1999) and ion trap instruments (Adhiya & Wesdemiotis, 2002; McLuckey et al., 2000), as well as during surface‐induced dissociation (SID) experiments (Mabud et al., 1985) where ions were activated via collision with a self‐assembled monolayer of fluoroalkanethiols on a gold surface (de Maaijer‐Gielbert et al., 1999). A much larger number of abundant fragments was produced in sustained off‐resonance irradiation collisional activation dissociation (SORI‐CAD) experiments (Gauthier et al., 1991) performed in FT‐ICR instruments (Han et al., 2005), due to a rather high amount of internal energy accumulated in the ions compared to CID. In contrast, only charge reduction was observed during ECD experiments reported in the same study, with the reduced [M + *n*H]^(*n*–1)+•^ odd‐electron ions formed upon capture of one electron readily ejecting H^•^ to generate the [M + (*n*–1)H]^(*n*–1)+^ even‐electron ions (Han et al., 2005). Performing SORI‐CAD on such ECD products permitted to conclude that the different protons adducted to PPI dendrimers have similar electron capture efficiency, regardless of their actual location in the dendrimer structure.

#### 2.2.3 MS/MS of polysulfonamide dendrimers

**Figure 14:**
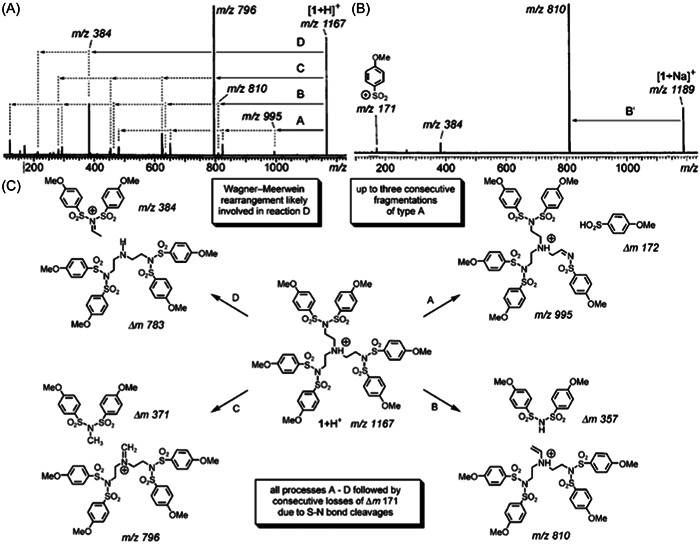
Collision‐induced dissociation spectra of polysulfonamide dendrimer G1 activated as (A) [M + H]^+^ or (B) [M + Na]^+^, and (C) the main four dissociation pathways experienced by protonated species. Adapted from Felder et al. (2005), with permission from John Wiley and Sons.

The large differences observed between CID spectra recorded for protonated molecules (Figure 14A) and alkali (Na or K) adducts (Figure 14B) of polysulfonamide dendrimers again illustrate how direct correlations can be established between fragmentation and charged sites (Felder et al., 2005). For dendrimers protonated at the core nitrogen atom, collisional activation induces four main fragmentation channels (Figure 14C). Release of 4‐methoxybenzene sulfinic acid (172 Da) in pathway A was proposed to occur via a 1,2‐elimination reaction and was indeed observed to proceed only once for each pair of branches. The least favored pathway B led to elimination of one 357 Da arm after 1,2‐proton transfer within the dendrimer core whereas the most abundant *m/z* 796 iminium fragment arose from pathway C. Finally, the *m/z* 384 formed after heterolytic cleavage of the C–NH^+^ bond in pathway D was proposed to be stabilized through a 1,2‐hydride shift. The far less populated MS/MS spectrum obtained for the sodiated dendrimer (Figure 14B) was explained by a dominant reaction induced by chelation of the alkali cation by two sulfonyl oxygen atoms followed by the release of one arm as a zwitterionic species, yielding a similar structure compared to the fragment obtained via pathway B in Figure 14C. In great contrast to these CID data, irradiation of sodiated sulfonamide dendrimers with a CO_2_ laser in infrared multiphoton dissociation (IRMPD) experiments was shown to result in a complex fragmentation pattern (Schubert et al., 2008), consistent with consecutive dissociation of primary fragments upon photon absorption (Sleno & Volmer, 2004). Three main reactions were reported: loss of SO_2_ through an *ipso* substitution, 1,2‐elimination of an alkene from the alkyl side chain at the focal point, and homolysis of N–S bonds, the latter process yielding radical species of different mass depending on the location of the dissociation center within the dendritic structure. In the same study, the authors also used MALDI‐TOF/TOF to reach higher collision regimes: accordingly, rearrangement processes were minimized while direct bond cleavages were favored (Schubert et al., 2008). Although fragmentation spectra acquired for different dendroisomers displayed distinguishable fingerprints, the authors acknowledged that the structure of an unknown isomer cannot be determined from its fragmentation pattern.

#### 2.2.4 MS/MS of poly(aminoester) dendrimers

**Figure 15:**
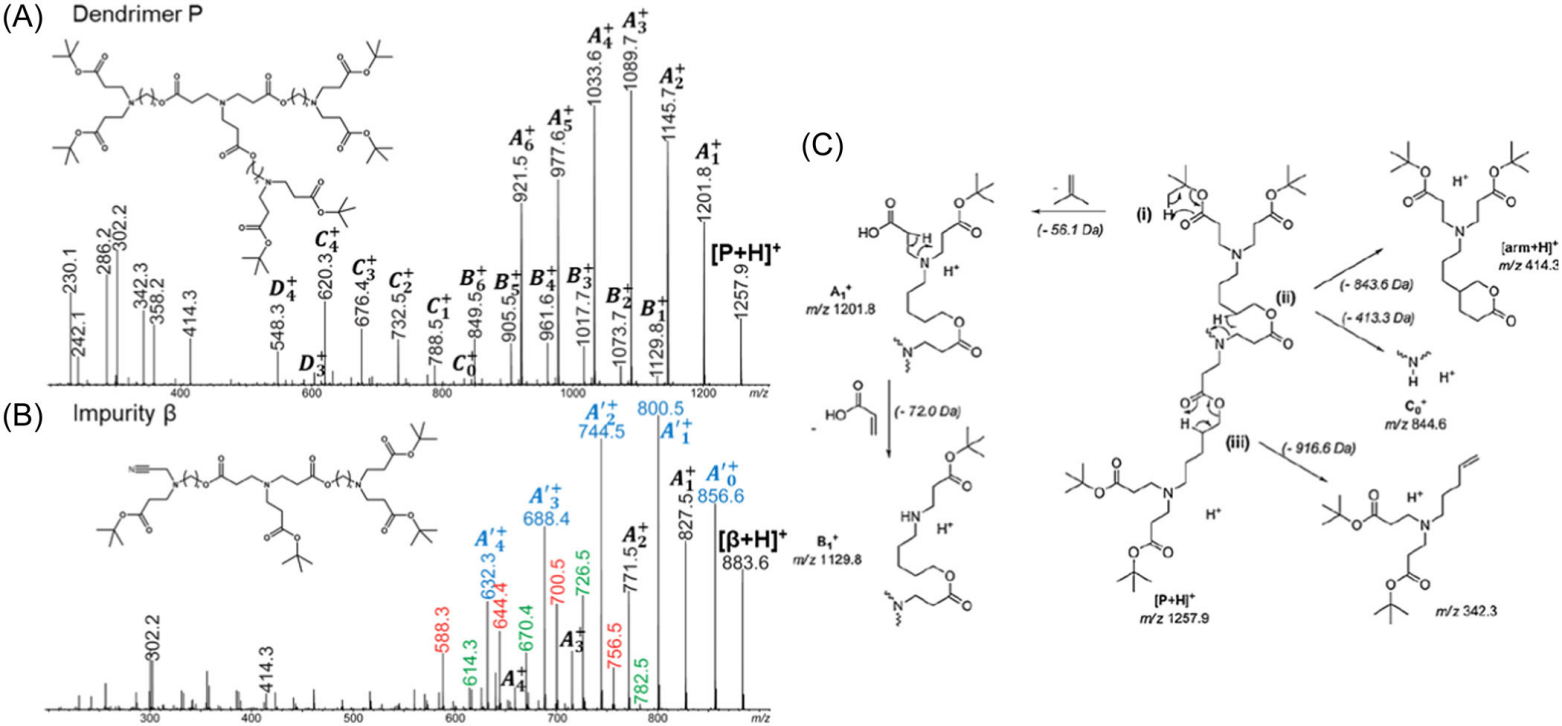
Collision‐induced dissociation spectra of (A) the G1 *t*‐Bu‐terminated poly(aminoester) dendrimer (P) and (B) a structurally related impurity (β). (C) Proposed mechanisms for the main dissociation reaction experienced by [P + H]^+^. Adapted from Tintaru et al. (2011), with permission from Elsevier B. V. [Color figure can be viewed at wileyonlinelibrary.com]

Mechanistic studies were performed for CID of poly(aminoester) dendrimers ended by acidic groups (Tintaru et al., 2010) as well as their precursors (named P) bearing *tert*‐butyl (*t*‐Bu) terminations (Tintaru et al., 2011). The latter P species were studied in the positive ion mode as protonated molecules and were observed to sequentially eliminate all their terminal *t*‐Bu as 2‐methylprop‐1‐ene to produce the A_i_^+^ series, with i indicating the number of these 56 Da losses (Figure 15A). This charge‐remote reaction (Figure 15C, pathway i) is observed to repeat in each branch and hence readily indicates the number of *t*‐Bu terminations in the dissociating precursor. The newly created acidic groups in so‐formed A_i_^+^ fragments are further released as acrylic acid (72 Da) to produce B_i_^+^ fragments. An alternative reaction from [P + H]^+^ was the detachment of an entire arm (Figure 15C, pathway ii), either released as a 413 Da neutral to produce the first member of the C_i_^+^ series or detected as a protonated species at *m/z* 414.3, both further eliminating all their *t*‐Bu moieties followed by 72 Da losses (the latter reaction accounting for the formation of D_i_^+^ fragments from C_i_^+^ ions). This dissociation behavior could be translated into useful rules to identify structurally related unknowns, as exemplified hereafter with the molecule named β which structure could not be intuitively derived from its Δ –374.3 Da mass defect. As shown in Figure 15B, the A_i_^+^ series comprises four members, hence indicating four *t*‐Bu end‐groups. Yet, as compared to the perfect dendrimer (Figure 15A), new major reactions were observed. The [β + H]^+^ precursor loses HCN to yield A′_0_^+^ at *m/z* 856.6, which reveals the presence of a cyano group. This A′_0_^+^ ion could eliminate up to four *t*‐Bu groups (blue series) but also, as supported by both accurate mass measurements and MS^3^ experiments, a 74 Da neutral (C_4_H_10_O) to form *m/z* 782.5 and a 100 Da neutral (C_5_H_8_O_2_) to form *m/z* 756.5. These new fragments eliminate up to three 56 Da neutrals, giving rise to the series respectively annotated in green and orange in Figure 15B. Because the new 74  and 100 Da losses were never observed from [β + H]^+^ or A_i_^+^ ions still containing the cyano group, it could be concluded that the CN moiety was located in a branch of an arm rather than in a branch directly connected to the central core. The structure proposed for β in Figure 15B to explain the two new reactions could be rationalized from the synthesis procedure (Tintaru et al., 2011).

The negative ion mode was best suited for ESI of poly(aminoester) dendrimers ended by acidic groups obtained after acidolysis of the six *t*‐Bu peripheral moieties in the dendritic precursor discussed in Figure 15A (Tintaru et al., 2010). The major CID reaction observed from these deprotonated dendrimers consisted of five successive 72 Da losses occurring from neutral carboxylic acid terminations: the eliminated neutral can either be acrylic acid, as depicted to occur from A_i_^+^ ions to yield B_i_^+^ in Figure 15C, or CO_2_ and ethane when considering a 1,5‐transfer of the acidic proton to the ternary nitrogen. Beside this major pathway, other dissociation reactions were quite similar to those observed for the *t*‐Bu‐terminated precursor, with successive detachment of two arms (–301 Da each) also observed as anions at *m/z* 300, and cleavage of one inner ester bond (–247 Da). All fragments still possessing neutral CH_2_CH_2_COOH branches further eliminated them as 72 Da neutrals. This fragmentation pattern could be translated into quite simple rules to readily identify defective molecules found in this sample, with the numbers of intact branches and unmodified arms in any selected precursors revealed by the number of iterative losses of 72 and 301 Da, respectively (Tintaru et al., 2010).

#### 2.2.5 MS/MS of polyether dendrimers

**Figure 16:**
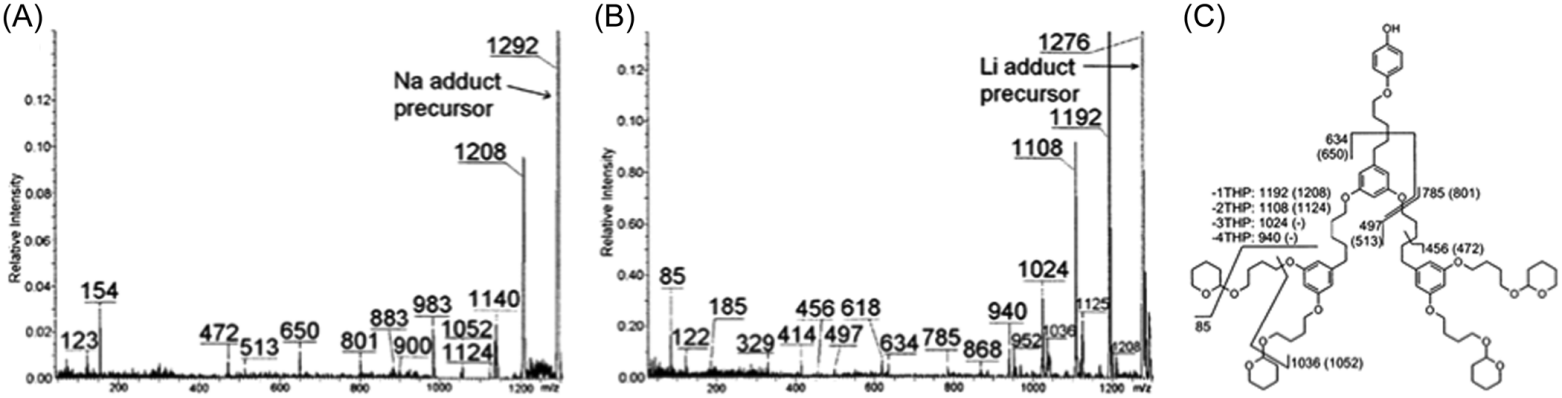
Matrix‐assisted laser desorption/ionization‐post‐source decay spectra of aryl ether‐like polypentylresorcinol dendrimer G2 adducted with (A) Na^+^ or (B) Li^+^ and (C) associated fragmentation scheme, with *m/z* of sodiated fragments into parentheses. Adapted from Neubert et al. (2003), with permission from the American Chemical Society.

Using the “hot” character of CHCA matrix for optimal PSD experiments, Neubert et al. showed that the fragmentation yield of aryl ether‐like polypentylresorcinol dendrimers strongly depends on the adducted alkali (Neubert et al., 2003). Similar to linear polyethers (Lattimer, 1992), release of the bare alkali cation was the main reaction observed from adducts containing large alkali (K^+^ and Cs^+^) whereas PSD data recorded for lithiated or sodiated species were the most informative for structural analysis of these dendrimers. More particularly, the –16 Da mass shift measured between nearly all product ions generated from [M + Na]^+^ (Figure 16A) and [M + Li]^+^ (Figure 16B) precursor ions was helpful to assign all detected fragments based on charge‐remote mechanisms summarized in Figure 16C. Such a dissociation pattern proved useful to obtain information about the number of end‐groups (via sequential losses of 84 Da dihydropyran) and regarding intact branching units, either eliminated as a 491 Da neutral to yield the sodiated *m/z* 801 (Figure 16A) or the lithiated *m/z* 785 fragment (Figure 16B), or detected as an alkali adduct at *m/z* 513 (Figure 16A) or at *m/z* 497 (Figure 16B). MS/MS data were also recorded for Fréchet dendrons with their benzyl bromide focal point converted to quaternary ammonium cation to enable positive mode ESI (Baytekin et al., 2009). Yet, these CID data were far less structurally useful since, although demonstrated to occur via a complex multistep cascade of rapid rearrangements, the main dissociation pathway ultimately yields a single fragment corresponding to the peripheral group.

### 2.3 IMS

IMS is a gas‐phase separation method in which ions are separated according to their mobility while traveling through a buffer gas under the influence of an electric field (Eiceman et al., 2013). In IMS, ions are dispersed according to their drift times (*t*_D_) in the mobility cell which are determined by their charge state and collision cross section (CCS), the latter being related to their three‐dimensional shape. As a postionization technique, IMS can be hyphenated to MS to perform a double separation dimension of gas‐phase ions, first as a function on their mobility then as a function of their *m/z* ratio (Bowers et al., 1993; Clemmer & Jarrold, 1997; Kanu et al., 2008). Accordingly, IMS‐MS coupling can be used either as a bidimensional separation technique for analytical purposes or as a tool to get detailed insight in the 3D structure of gas‐phase ions by comparing experimental CCSs with theoretical values calculated from 3D models. Although both options are very appealing for dendrimers, IMS is still not widely applied in the field. Yet, a few studies can be found in the literature and, not surprisingly, they mostly investigate the archetypal PAMAM dendrimer model. These studies all employed the same IMS technology, namely traveling wave ion mobility spectrometry (TWIMS) (Giles et al., 2004; Giles et al., 2010). In this device, ions with larger CCS experience more collisions with the buffer gas and have their mobility reduced compared to more mobile ions of lower CCS that hence travel faster through the cell. The TWIMS device operates at low but constantly changing electric field and, as such, needs to be calibrated using t_D_ values of calibrants with known CCS (Ruotolo et al., 2008). In this instrument, analysis of ions is ultimately performed with a TOF mass analyzer.

For analytical purposes, the TWIMS cell can also be operated in tandem with a mass selective analyzer (i.e., a quadrupole) and a collision cell, as illustrated in a first study where IMS was used as a time‐dispersive method to resolve defective PAMAM structures with different charge states but exactly the same *m/z* value (Maire et al., 2013). As previously discussed, ESI most often distributes species over multiple charge states, which can give rise to severe signal overlaps in mass spectra recorded for samples containing impurities. This was the case for a PAMAM sample where the singly charged G0 molecule with one molecular loop (456.3 Da), the doubly charged G1 lacking four arms and containing one molecular loop (916.6 Da) and the triply charged G1 with one molecular loop (1369.0 Da) were all detected at the same monoisotopic *m/z* 457.3 value. Such *m/z* coincidences obviously prevent proper MS/MS experiments since precursor ion selection cannot be done for individual species. Performing IMS before MS analysis allowed the three *m/z* 457.3 ionic species to be sufficiently separated for individual injection in the collision cell placed after the TWIMS device, hence enabling safe MS/MS structural characterization.

**Figure 17:**
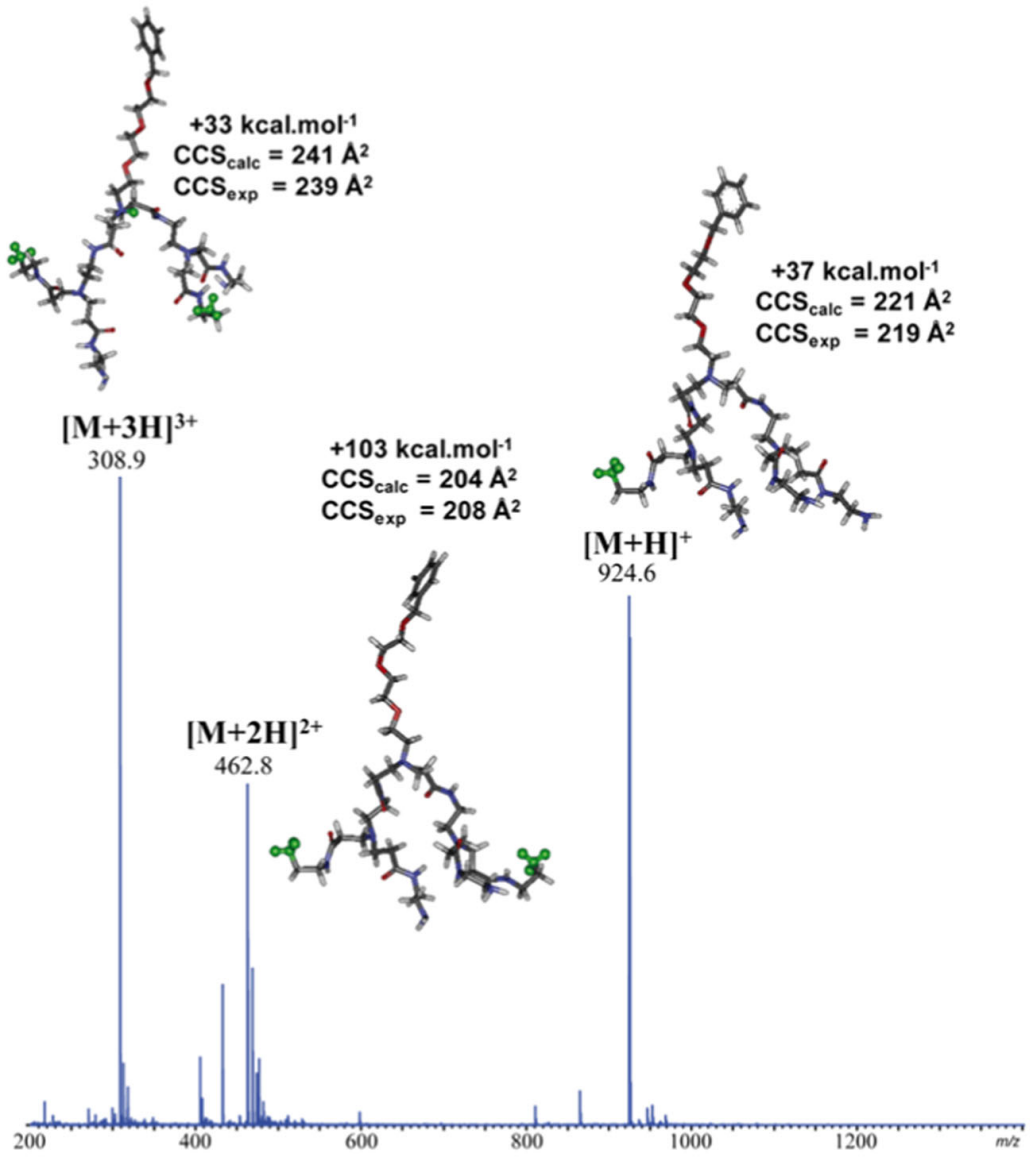
Positive mode electrospray ionization mass spectrum of the fan‐shape poly(amidoamine) dendrimer with inset structures showing the most stable conformations (with protonated groups in green) found for each charge state, with their potential energy (in kcal mol^−1^), calculated CCS (CCS_calc_, in Å^2^) and experimental CCS (CCS_exp_, in Å^2^). Adapted from Tintaru et al. (2013), with permission from Elsevier B. V. [Color figure can be viewed at wileyonlinelibrary.com]

**Figure 18:**
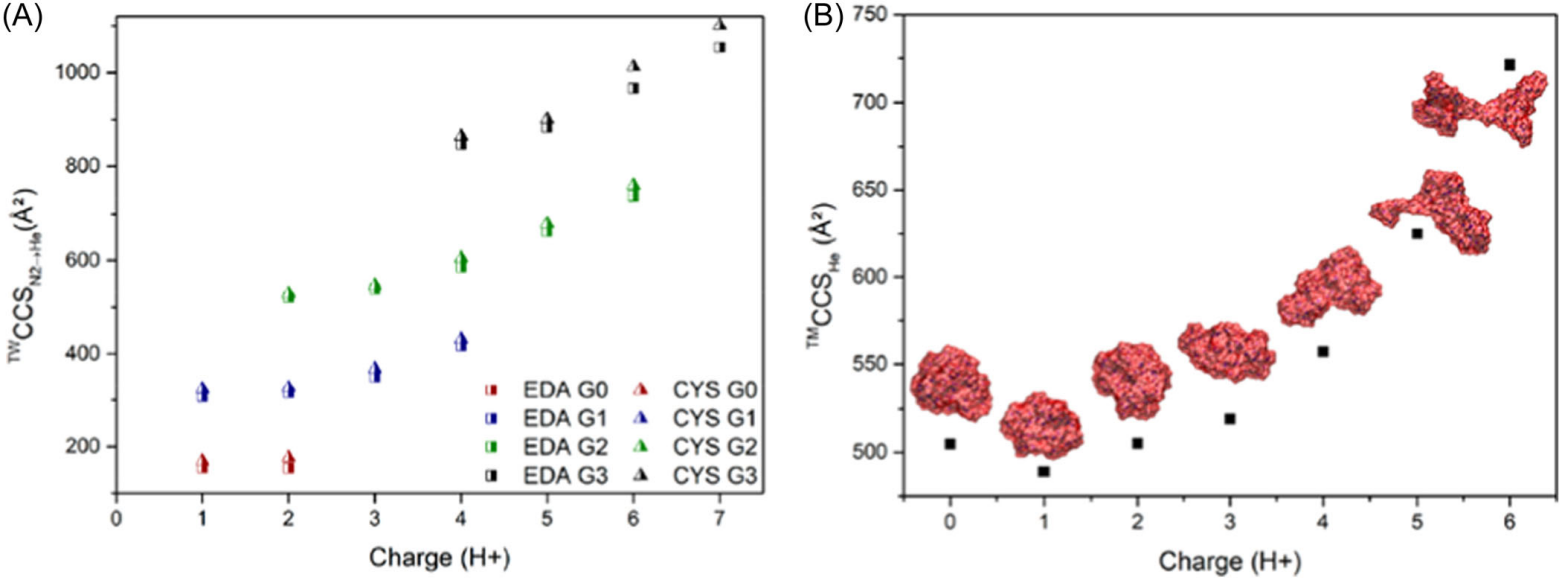
(A) Evolution of the experimental collision cross section (CCS) of ethylene diamine (EDA)‐ and cystamine‐core poly(amidoamine) (PAMAM) (G0–G3) as a function of their protonation state. (B) Evolution of the shape and associated CCS of EDA‐core PAMAM G2 as a function of its charge state ranging from 0 to 6. Adapted from Saintmont et al. (2020), with permission from the American Chemical Society. [Color figure can be viewed at wileyonlinelibrary.com]

Coupling IMS to MS also provides valuable information regarding the 3D structure of dendrimer ions and to address the question of how charge distribution within the structure changes its conformation. It should be noted that the role of the solvent is no longer considered in these techniques operating on gas‐phase species. As a result, so‐obtained data are not expected to be directly transposable to solution phase phenomena but, instead, should shed light on the intrinsic behavior of investigated species. In a first study, changes of molecular conformation were conjectured from different charge state distributions observed in ESI‐MS for a fan‐shape PAMAM grown from a tri(ethylene glycol) segment as compared to a spherical PAMAM built from a triethanolamine (TEA) core (Tintaru et al., 2013). The TEA‐core PAMAM G1 exhibited a classical monotonic charge state distribution, with major signals for the doubly (100%) and triply (62%) protonated molecules while the abundance of the singly charged dendrimer was below 5%. In great contrast, the fan‐shape PAMAM preferentially ionized with 1 or 3 protons while the 2+ charge state was disfavored (Figure 17), suggesting conformational changes as a function of the number of protonated sites. This peculiar distribution was supported by potential energy values calculated for the most stable conformations found by molecular dynamics simulations (inset structures of Figure 17). It was found that primary amine end‐groups readily interact with charges whereas the last group to be protonated was the central tertiary amine. CCS calculated for these most stable conformations (inset of Figure 17) were consistent with experimental CCS values derived from drift times of the three ions: the hydrodynamic volume of the fan‐shape dendrimer slightly decreased (–5%) when increasing the charge state from 1+ to 2+, while addition of a third proton induced a significant swelling (+15% CCS increase) of the molecules. According to theoretical calculations, these conformational changes were dictated by electrostatic repulsions. As compared to the fan‐shape dendrimer, volume contraction induced by adduction of a second proton on the globular TEA‐core PAMAM G1 was negligible but the swelling phenomenon was much more pronounced (+25% CCS increase) when reaching the 3+ charge state (Tintaru et al., 2013). A similar trend was observed when studying CCS variation as a function of the number of charges over different generations (G0–G3) of PAMAM dendrimers with alternative cores, namely ethylene diamine (EDA) (Maire et al., 2013; Saintmont et al., 2020) or cystamine (CYS) (Saintmont et al., 2020). Nearly identical CCS values were measured for the two lowest charge states adopted by each generation whereas CCS increased significantly as the protonation degree further increased (Figure 18A). These data were used to further question the globular shape of dendrimers as a function of their charge state by studying deviation of experimental points from the CCS = A.M^2/3^ trend expected for spherical objects (with M the mass and the A coefficient reflecting the ion density). As supported by molecular modelling and illustrated in Figure 18B for PAMAM G2, globular shapes were found for the lowest charge states whereas the dendrimer shape gets more and more elongated upon stepwise addition of protons, with maximal separation of the branches observed for the 6+ ions (Saintmont et al., 2020). The same trend was observed independently of the core structure (EDA vs. CYS) and the PAMAM generation. This study also includes similar analyses for PPI dendrimer ions, found to be slightly more packed (0.13 atoms/Å^3^) than protonated PAMAMs (0.12 atoms/Å^3^).

## 3 DENDRIMERS FOR MS

Complementary to benefits of MS for structural characterization of dendrimers as reviewed in Section 2, we wished to focus here on benefits offered by dendrimers in the field of MS. Their structural diversity and well‐defined structure over multiple generations are indeed appealing features to employ dendrimers in the development of MS methods. Even properties found to be serious limitations for their analysis can be turned into advantages when considering them as chemical reagents. For example, the potential of some dendrimers to act as energy‐absorbing surface is an issue for their MALDI analysis but becomes an essential property for their use as MALDI matrices (Neubert et al., 2002). However, it should be acknowledged that, besides specific chemical or physical properties, the key point for dendrimers to become popular as standards or reagents remains their commercial availability. This is the reason why only two main dendrimer families, namely PAMAMs and (bis‐MPA)‐based dendrimers, are reported in the literature for their analytical benefits when used as standards for mass calibration (in MS) or size calibration (in IMS) on the one hand, and as reagents for gas‐phase ion–ion reactions on the other hand.

### 3.1 Dendrimers as standards for calibration

Auspicious advances in mass analyzer technologies have led to substantial improvements in terms of resolving power, enabling elemental composition of ions to be derived from their *m/z* value providing proper calibration to ensure a high degree of mass accuracy. In particular, when investigating unknown structures, accurate and precise mass assignment are paramount. Consequently, the continuous advancement of MS relies on the paralleled progress of mass calibrants that are simple to use and cost‐efficient but also comprised of robust species that cover a broad mass range and have extended shelf‐lives at ambient conditions. When dealing with internal mass calibration, standards should also be free of contaminants, and their signals, aimed at bracketing unknown peaks, should be sufficiently spaced to prevent interferences but close enough to provide accurate mass calibration. To keep sample preparation simple, internal standards should also have sufficient versatility to be compatible with experimental conditions optimized for analyte ionization, such as solvents or salts in ESI or matrix in MALDI. These constraints are hardly addressed with standards commonly used for mass calibration, such as mixtures of peptides or proteins, polydisperse synthetic polymers, or ion clusters. On the one hand, biomolecule standards suffer from poor signal resolution, restricted matrix compatibility, and limited selection of masses especially in the high molecular weight domain, which makes it difficult to obtain an accurate calibration curve spanning a wide *m/z* range. In addition to their cost and need for extensive purification, the greatest problem plaguing peptide and protein standards is their inherent instability in bulk and in solution, with a high propensity to the formation of defective structures that may lead to peak misidentification that generates inaccurate mass calibration. On the other hand, synthetic polymers and ion clusters offer an inherent mass polydispersity usefully employed for multiple‐point calibration. Yet, although attractive in terms of cost and shelf‐lives, these alternatives are most useful for low‐mass calibrations. In addition, presence of impurities in synthetic polymer samples or low‐mass repeating units defining small peak spacing may raise issues in terms of analyte signal overlap. In ESI‐MS, potential memory effects experienced with popular standards such as poly(ethylene glycol) limit analytical throughput (Konig & Fales, 1999) while limited solubility of synthetic polymers in many solvents as well as their matrix‐dependent ionization are two major issues when using MALDI. Popularity of inorganic ion clusters such as CsI clusters relies on their lack of isotopic pattern, which limits potential signal interference and avoids erroneous peak picking. However, formation of such clusters in both ESI and MALDI most often requires source parameters significantly different from those required for analytes.

**Figure 19:**
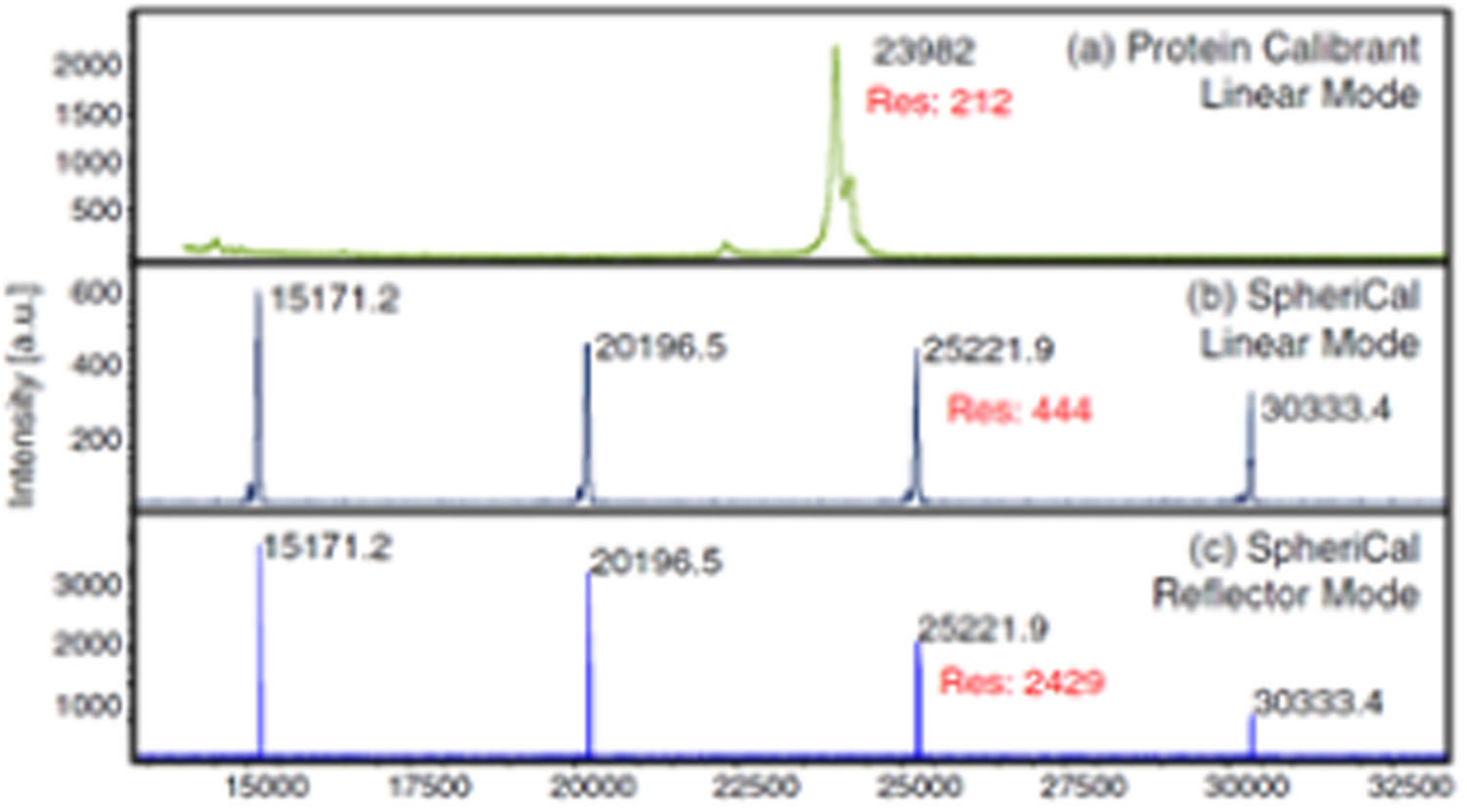
As compared to (A) commercial proteins such as trypsinogen typically used as matrix‐assisted laser desorption/ionization mass standards, mixture of bis‐MPA‐based dendrimers as found in SpheriCal standards enables mass calibration of a wider *m/z* range in both (B) linear and (C) reflectron mode time of flight. Reprinted from Grayson et al. (2014), with permission from the American Chemical Society. [Color figure can be viewed at wileyonlinelibrary.com]

Some dendrimers offer the potential to combine all advantages of these traditional standards while avoiding their drawbacks. This is the case of polyester‐based dendrimers recently proposed as alternative standards for mass calibration and made commercially available as SpheriCal®. These bis(hydroxymethyl)propanoic acid (bis‐MPA)‐based dendrimers were selected based on the inherent purity of their divergent synthetic route, a coupling of highly efficient esterification and deprotection reactions that are easily driven to completion without detectable side reactions (Grayson et al., 2014). These truly monodisperse samples can be obtained over a broad mass range (up to 30 kDa) (Giesen et al., 2018; Grayson et al., 2014) and can be intentionally mixed to generate evenly spaced well‐defined peaks across defined *m/z* ranges, enabling improved calibration compared to single‐point standard such as in protein calibrant (Figure 19A) in both linear (Figure 19B) and reflectron (Figure 19C) mode TOF. These standards are available as either individual compounds or premixed kits covering different mass ranges, for example, *m/z* 3500–7500, *m/z* 7500–15,000, or *m/z* 15,000–31,000. To obtain multiple calibration points at each generation, various cores were used. When designed for MALDI, these kits come in a ready‐to‐use mixture with a matrix, sodium trifluoroacetate (NaTFA) salt, and four dendrimers from Sigma‐Aldrich and Polymer Factory (Gross, 2016). As previously detailed in Section 2.1.8, these dendrimers exhibit an exceptionally broad compatibility with a wide range of matrices and solvents (Giesen et al., 2018; Grayson et al., 2014). Accordingly, SpheriCal® kits are available with different matrices including 9‐NA, CHCA, HPBA, DCTB, SA, or 2,5‐DHB. As demonstrated by Gross, the quality of mass calibration with SpheriCal® dendrimers can further be improved thanks to (i) enhanced signal resolution at low laser fluence by using a binary mixture of DCTB and 9‐Na and (ii) increased number of calibration points by simple addition of a second salt in the sample to favor simultaneous production of different cation adducts (Gross, 2016).

**Figure 20:**
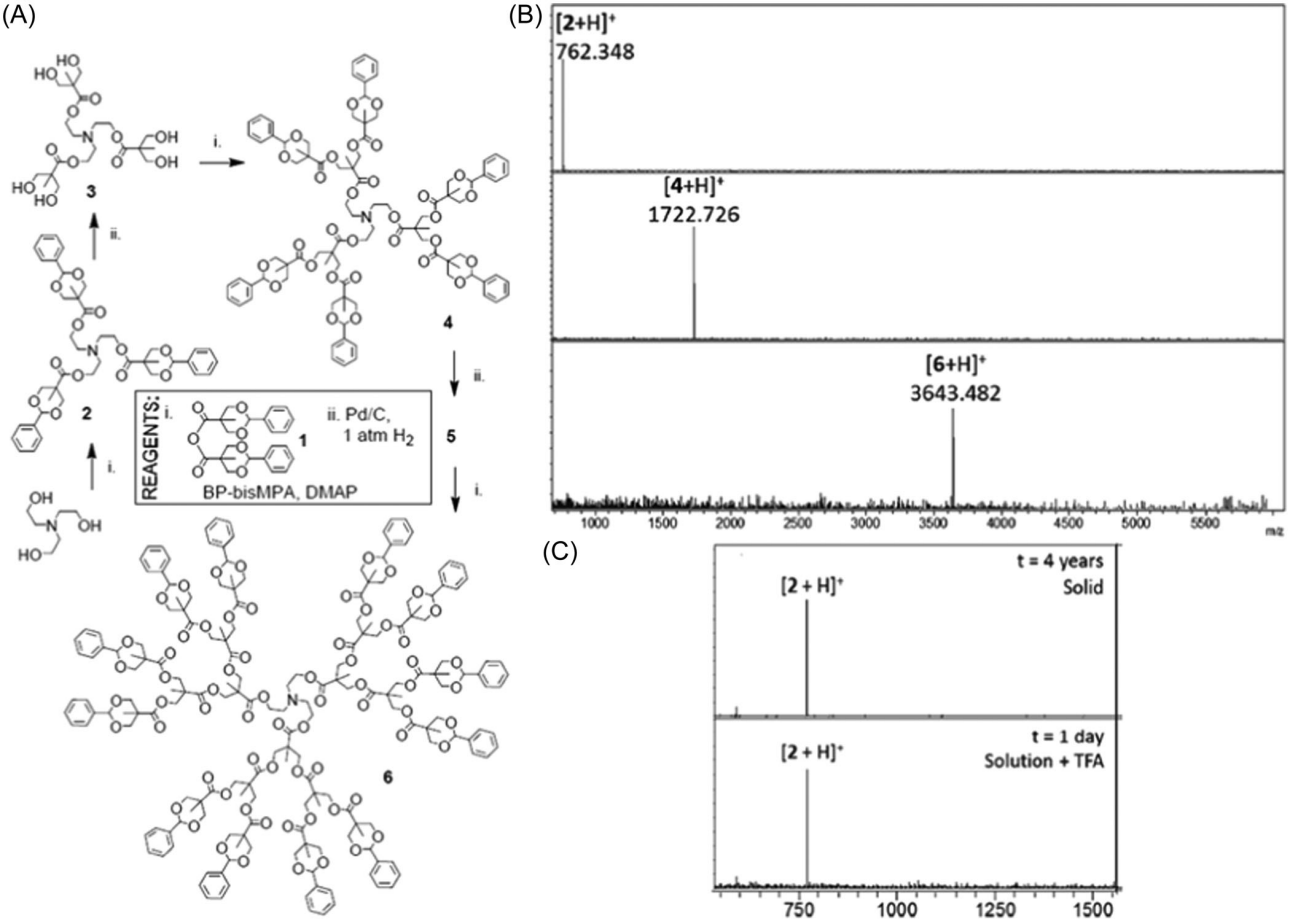
(A) Synthesis of triethanolamine‐core bis(hydroxymethyl)propanoic acid dendrimers G1–G3 to enable, (B) protonation upon matrix‐assisted laser desorption/ionization (MALDI) (matrix: FA, 0.1% trifluoroacetate [TFA]). (C) Stability study: MALDI mass spectra of the G1 dendrimer stored for 4 years as a solid (top) or for 24 h in a 0.1% TFA solution (bottom). Adapted from Casey and Grayson (2015), with permission from SAGE Publications.

Another major advantage of SpheriCal® dendrimer calibrants is their exceptional shelf‐life at room temperature. To test the loss of performance of the calibrants in multiple conditions, Grayson et al. compared calibrant mass data obtained with a pure dry powder, a powder premixed with matrix (9‐NA), and a solution with salt and matrix in acetonitrile (Grayson et al., 2014). The dry powder samples showed stability for over 3 years (when the paper was published) and 11 years now, which is far longer than any traditional protein or peptide standards. The solution displayed stability for longer than 1 month, and even longer when prepared in the least polar solvents. Indeed, in contrast to their linear analogues, bis‐MPA‐based dendrimers exhibit enhanced solubility across a wide range of solvents thanks to their globular shape and highly branched structure. The same group established that calibration data of high quality can be readily obtained by spotting samples from solutions made in hexane, dichloromethane, chloroform, ethyl acetate, acetone, methanol, ethanol, acetonitrile, dimethyl sulfoxide, and some aqueous solutions. The 100% water conditions could not be tested due to the lack of matrix solubility for the benzylidene dendrimers. However, their deprotected version holding hydroxy end‐groups are water‐soluble and can hence be used as standards in polar solutions (Grayson et al., 2014). Due to their solubility in numerous solvents, bis‐MPA‐based dendrimers are also available as SpheriCal®‐ESI calibrants. Once electrosprayed, they show intense signals with no severe memory effect even at low infusion rates, and their compact structure favors the formation of singly charged species whereas adduction with multiple cations only occurs for the highest generations prone to screen charge repulsion (Romson et al., 2021). As evaluated by Romson and coworkers when analyzing a cytochrome *C* digest, the SpheriCal®‐ESI mix was found to perform better than the most traditional ESI Tuning Mix, not only in terms of identified peptides (18 based on 35 *m/z* values vs. 16 based on 26 *m/z* values) but also in terms of accuracy and precision (Romson et al., 2021). It is to be noted that bis‐MPA‐based dendrimers have better hydrolytic stability than esters usually do (because all of these are pivalate esters) especially in acidic conditions, which makes them suitable for use with most peptide and protein analytes. For this purpose, Casey and Grayson took advantage of the inherent modularity of dendrimer synthesis to modify the core of bis‐MPA‐based species and hence increase their proton affinity (Casey & Grayson, 2015). Although containing a single amine group at the core (Figure 20A), these modified dendritic calibrants were observed to readily protonate (Figure 20B) and, like the classical SpheriCal® dendrimers, they exhibit a lengthy shelf‐life (Figure 20C), high solvent compatibility and strong matrix compatibility.

**Figure 21:**
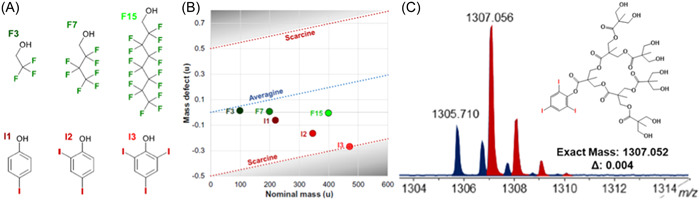
(A) Fluorinated or iodinated alcohols considered as potential dendrimer cores and (B) plot of their mass defect versus their nominal mass from the averagine to the “scarcine” valley defined for peptides. (C) Matrix‐assisted laser desorption/ionization‐time‐of‐flight mass spectrum of a BSA peptide (frag 402–412, in the dark blue) with the triiodo [G3]‐(OH)_8_ calibrant (in red). Adapted from Giesen et al. (2018), with permission from the American Chemical Society. [Color figure can be viewed at wileyonlinelibrary.com]

An alternative strategy to improve the relevance of dendrimers for mass calibration is the use of mass‐defect labels to tag calibrants to limit the likelihood of the internal standards to overlap with analyte signals across the entire desired *m/z* range. Since most biological and synthetic macromolecules (almost exclusively composed of H, C, N, and O) all exhibit a mass defect that is either positive or negligibly negative, ideal internal standards should hence have a clearly negative mass defect. To identify best initiators for dendrimer‐based mass‐defect calibrants, Grayson and coworkers first made a database of the calculated mass‐defect distributions for the 20 most common proteinogenic amino acids (Giesen et al., 2018). Then, fluorinated and iodinated cores (Figure 21A) were explored to determine which core would have a mass defect furthest from the averagine where the majority of the peptide signals appear (Figure 21B). Because iodine exhibits a negative mass defect nearly 60 times greater than fluorine, iodinated aromatic cores clearly appeared as the best candidates. More particularly, the tris‐iodinated aromatic core located directly in the “scarcine” valley (0.5 *m/z* from the averagine), which is the area with the least number of potential analyte signals, was selected to prepare triiodo[G3]‐(OH)_8_ dendrimer standard. As illustrated in Figure 21C, the calibrant (in red) is clearly resolved from the peptide analyte (in blue) despite the fact that their isotopic distributions overlap. Moreover, the isotopic signature of the tri‐iodinated dendrimer largely contrasts that of peptides, hence enabling unambiguous distinction between standard and analyte signals.

The robust shape of dendrimers should also be turned into a clear advantage for IMS calibration but, so far, only one report was found in this area, with PAMAM dendrimers used as standards for differential mobility analysis (DMA) (Imanaka et al., 2006). DMA has become very popular to perform in situ measurements of nano‐size particles, which becomes increasingly challenging as the particle size decreases, in particular with regard to instrument calibration. As measured by alternative techniques such as small angle X‐ray scattering (SAXS) (Prosa et al., 1997), PAMAM dendrimers were considered as relevant candidates to fill the gap between the low range limit of polystyrene spheres (20 nm) and the high range limit of atomic clusters such as C_60_ (1 nm) (Mulholland & Bauer, 2000). Using ESI to generate gas phase ions of PAMAM G2–G6 in both positive and negative ion modes, Imanaka et al. showed that these spherical molecular particles have a diameter ranging from 2.45 to 5.80 nm, with no significant difference between the two polarity modes (Imanaka et al., 2006). These values happen to be 20%–30% smaller than diameters determined by SAXS, as often reported for gas‐phase conformations observed to be more compact than conformations in solution. It should however be noted that the experimental set‐up used in this study did not include any mass measurement to support proper assignments of the two peaks systematically observed for all PAMAM samples in mobility spectra. In contrast, the robustness of protonated PAMAM and PPI structures assessed by coupling MS with IMS as well as the good agreement between calculated and experimental CCS makes these dendrimers suitable calibrants for TWIMS (Saintmont et al., 2020).

### 3.2 Dendrimers as reagents for gas‐phase reactions

**Figure 22:**
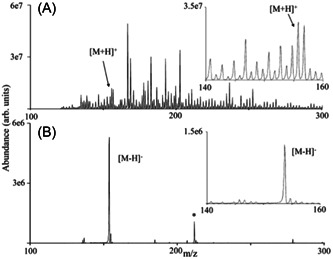
(A) Positive electrospray ionization mass spectrum of a precipitated plasma solution spiked with histidine (100 μM). (B) Negative mode mass spectrum obtained after ion/ion reaction with [PAMAM‐G1.5 – 6H]^6–^ reagent anions. Reprinted from Hassell et al. (2011b), with permission from the American Chemical Society.

Gas‐phase ion/ion reactions enable manipulation of the charge state as well as the polarity of electrosprayed ions independently of the initial ionization conditions. This can be particularly useful in the field of peptides, when structural information available from ion dissociation are sensitive to the precursor charge state, or when MS/MS of ions or opposite polarity can provide complementary information. Such experiments are performed with ion trap mass spectrometers, with modifications allowing for the sequential injection (and subsequent reactions) of oppositely charged species generated from two different ESI sources (Badman et al., 2002; Wells et al., 2002). Typically, positive ions are accumulated in the ion trap, specific charge is isolated, then anions serving as charge inversion reagents are directed into the ion trap where the ion/ion reaction is allowed to occur for define periods of time. Alternatively, the process can also be envisaged with negatively charged analytes and positively charged reagents. For charge inversion of species, use of multiply charged reagents is a more efficient approach than a sequence of ion/ion reactions where each step permits to change one charge only. Moreover, due to technical issues limiting the *m/z* range over which ions of opposite polarities can be stored in ion traps, charge inversion of high‐mass peptides requires the use of high‐mass anionic reagents. In this context, carboxylate‐terminated half‐generation PAMAM dendrimers were found to be particularly valuable reagents as they allow charge inversion to occur via proton transfer rather than via adduct formation. The former process, changing [M + nH]^*n*+^ ions into [M – mH]^m–^ products, is indeed more appealing since dissociation of deprotonated products are far more structurally informative compared to the R neutral losses observed when activating the [(M + nH)•(R – *x*H)]^(*x*−*n*)–^ adduct (with *x* > *n*). For example, deprotonated ubiquitin with charge state up to 4– could be produced when leaving the singly protonated molecules react in the gas phase with deprotonated G1.5 to G3.5 PAMAM, while adduct formation was only observed to proceed to a very low extent with the G0.5 anionic reagent (He et al., 2005). The strong charge inversion capability of the carboxylate‐terminated half‐generation PAMAM offers clear analytical benefits. It was used to convert the multiple cationic forms that dilute the analyte signal into a singly deprotonated molecule (Hassell et al., 2009). It also enabled drastic chemical noise reduction in mass spectra (Hassell et al., 2011b): as compared to the noisy data shown in Figure 22A where signal of protonated histidine is hardy observed (signal‐to‐noise ratio <2), the negative ion spectrum obtained after ion/ion reaction with [PAMAM‐G1.5 – 6H]^6–^ reagent anions displays a dominant (signal‐to‐noise ratio > 200) signal for deprotonated histidine (Figure 22B). These data indicate that the large majority of cations composing the chemical background have been neutralized whereas the protonated amino acid has efficiently undergone two proton transfer reactions.

As gas‐phase reagents, these half‐generation PAMAMs also allowed development of a novel type of charge inversion reaction consisting of cation/anion substitution on the analyte (Hassell et al., 2011). In this case, the dendrimer was used for its propensity to form multiply charged anions able to remove one cation from the analyte, but also for its ability to host, in its pocket‐like structure, small anions to be transferred to the analyte molecule. Such a multifunctional charge inversion reagent was created by electrospraying a solution containing PAMAM‐G3.5 and Cu(NO_3_)_2_ and used to switch the protonated beclomethasone steroid into a [M + NO_3_]^–^ species upon ion/ion reaction. Noteworthy, the method permitted to distinguish corticosteroids, with high affinity towards anions and no acidic sites, from estrone which does not meet these two requirements for ultimate formation of anionic adduct. Full generation amino‐terminated PAMAM have also been reported as efficient charge permutation reagents to convert small amphiprotic molecules from their protonated to their deprotonated state (Hassell et al., 2011a, 2011). Similar to PAMAM, DAB‐PPI dendrimers were reported as efficient proton donors in ion/ion reactions without appreciable adduct formation. For example, multiply protonated DAB‐PPI(G4) was used to turn deprotonated polypeptides into protonated species (Emory & McLuckey, 2008, 2009). This study also showed that the number of protons to be transferred can be conveniently tuned by playing with both DAB generation number and charge state. Accordingly, [DAB‐PPI(G4) + 7H]^7+^ was involved in ion/ion charge inversion of deprotonated phosphopeptides to generate doubly protonated products, which are particularly hard to form in positive mode ESI when in mixture with basic peptides but of high interest since they are amenable to electron‐transfer dissociation (Gunawardena et al., 2006). Finally, PAMAM and DAB‐PPI can be combined in sequential ion/ion reactions to increase ion charge state. Negatively charged PAMAM‐G0.5 was used in conjunction with positively charged DAB‐PPI(G4) for a double charge inversion aimed at increasing the number of protons on protonated bradykinin, using the former for charge reduction of the peptide from 1+ to 1– while the latter was involved in a second ion/ion reaction to enable the charge state to be switched from 1– to 2+ (He & McLuckey, 2003). Changing the reagent sequence permitted to increase the negative charge of peptide and oligonucleotide macroanions, first changed from their initial 1– charge state to singly protonated species upon 700 ms reaction with protonated DAB‐G4, then observed as [M – 2H]^2–^ after a second 700 ms reaction step involving multideprotonated PAMAM‐G3.5 (He & McLuckey, 2004a).

## 4 CONCLUSIONS AND PERSPECTIVES

MS proved to play a significant role in dendrimer chemistry and has hence become a routine technique in the field. MS enables rapid determination of sample purity even for higher generations while MS/MS allows unambiguous identification of defective structures, a mandatory step towards rational improvement of dendrimer synthesis. The vast majority of studies reviewed herein employed ESI or MALDI to produce gas‐phase ions but, on this side, no universal recipe (notably regarding the choice of best MALDI matrices) is to be expected for dendrimers since ionization characteristics highly depend on the analyte chemistry. In this context, for those dendrimers with terminating groups playing a major role during the ionization step, it can be anticipated that surface modification performed to achieve advanced performance (Duran‐Lara et al., 2015) may require new experimental conditions to be developed for proper ionization of postfunctionalized species. Alternatively, when ion formation is dictated by the chemistry of the dendrimer skeleton, significant modifications of the ionization behavior were reported for amphiphilic dendrimers (Wang & Grayson, 2012) or dendrimer conjugates (Tintaru et al., 2014) developed for their unique properties in the field of drug delivery. Similar remarks also apply for MS/MS experiments, with fragmentation routes relying highly on dendrimer chemistry, ionic forms of the dissociating species and activation methods. Notably, the exceptional variety of activation techniques employed for dendrimers clearly highlights relevance of these well‐defined macromolecules as analyte models, complementarily to the major benefits they offer in the development of MS methods discussed in Section 3. As previously mentioned, this review article was purposely restricted to organic dendrimers as obtained at the end of their synthesis and, as such, it does not fully reflect the dynamism of this research field nor the capabilities of MS‐based techniques. In particular, the coupling of MS with IMS offers advantageous promises to study dendrimer‐based complexes, with ESI ensuring their gas phase transfer as intact species, MS readily revealing their stoichiometry and IMS enabling detailed understanding of their conformational dynamics, as recently reported for giant metallodendrimers (Xie et al., 2017) and dendriplexes (Saintmont et al., 2022).

## AUTHOR CONTRIBUTIONS

**McKenna J. Redding**: Writing—original draft. **Scott M. Grayson**: Conceptualization; writing—original draft. **Laurence Charles**: Conceptualization; validation; writing—original draft; writing—review and editing.
